# Seasonal environmental variability drives microdiversity within a coastal Synechococcus population

**DOI:** 10.1111/1462-2920.15666

**Published:** 2021-07-26

**Authors:** Kristen R. Hunter‐Cevera, Bryan R. Hamilton, Michael G. Neubert, Heidi M. Sosik

**Affiliations:** ^1^ Josephine Bay Paul Center Marine Biological Laboratory Woods Hole MA USA; ^2^ Biology Department Woods Hole Oceanographic Institution Woods Hole MA USA

## Abstract

Marine microbes often show a high degree of physiological or ecological diversity below the species level. This microdiversity raises questions about the processes that drive diversification and permit coexistence of diverse yet closely related marine microbes, especially given the theoretical efficiency of competitive exclusion. Here, we provide insight with an 8‐year time series of diversity within *Synechococcus*, a widespread and important marine picophytoplankter. The population of *Synechococcus* on the Northeast U.S. Shelf is comprised of six main types, each of which displays a distinct and consistent seasonal pattern. With compositional data analysis, we show that these patterns can be reproduced with a simple model that couples differential responses to temperature and light with the seasonal cycle of the physical environment. These observations support the hypothesis that temporal variability in environmental factors can maintain microdiversity in marine microbial populations. We also identify how seasonal diversity patterns directly determine overarching *Synechococcus* population abundance features.

## Introduction

Approximately 60 years ago, G. E. Hutchinson posed the question: how do thousands of different phytoplankton species simultaneously coexist in a seemingly uniform aquatic environment (Hutchinson, 1961)? In other words, how is it that one species does not come to dominate or out‐compete all others in a system that (at first glance) appears to be limited in the environmental dimensions available for differentiation. This question has captivated scientists since it was proposed, and many researchers, including Hutchinson himself, have contributed theory and observations to help explain this apparent paradox (Roy and Chattopadhyay, 2007).

A magnified version of this paradox is the diversity that can be found within a group of organisms that are very closely related to one another, often termed *microdiversity* (Acinas *et al*., 2004). The marine cyanobacteria *Prochlorococcus* and *Synechococcus* are widespread and important primary producers that contain such microdiversity (Scanlan *et al*., 2009). These two groups are genetically partitioned into several different clades, and these genetic delineations often reflect distinct ecologies and physiologies. Clades differ in light‐harvesting capability (Biller *et al*., 2015), chromatic adaptation and pigment composition (Palenik, 2001; Ahlgren and Rocap, 2006), nutrient utilization (Moore *et al*., 2002), temperature growth responses (Johnson *et al*., 2006; Pittera *et al*., 2014) and other attributes.

Clades also differ in their biogeography, and much of our understanding about picocyanobacteria diversity has been informed by biogeographical studies. This is especially true of *Synechococcus*, where niches have mainly been inferred from where clades have been observed in the ocean. For example, certain clades are only found in cooler and more nutrient‐rich waters, whereas others tend to occur in warm oligotrophic waters (Zwirglmaier *et al*., 2007; Sohm *et al*., 2016).

While spatial explorations have provided insight into the environmental factors that may govern diversity patterns, temporal variability is an important driver of *Synechococcus* diversity. Studies that have investigated diversity over time show that clade composition is typically not constant over a year and that changes in environmental conditions result in changes in relative abundance or even succession patterns (Tai and Palenik, 2009; Post *et al*., 2011; Ahlgren *et al*., 2019; Larkin *et al*., 2020).

At the Martha's Vineyard Coastal Observatory (MVCO), *Synechococcus* population dynamics are governed by seasonal environmental changes (Hunter‐Cevera *et al*., 2016a, 2020a). The annual cycle of cell concentration varies from a few hundred cells ml^−1^ in winter to up ∼10^5^ cells ml^−1^ at the start of summer. Cell division rates are temperature‐limited in winter and into spring but become light limited at the beginning of fall. Seasonal cell abundance patterns result from these physiological limitations on growth combined with population losses from either protist grazers or viral lysis. These population dynamics, however, are not the consequence of only one type of *Synechococcus* responding uniformly to a changing environment. We have documented significant diversity within the population; at least 13 different clades at MVCO have been identified from clone libraries and culture isolations (Hunter‐Cevera *et al*., 2016b).

To gain insight into how this microdiversity determines abundance dynamics of the *Synechococcus* population and how such diversity is maintained at MVCO, we leverage an 8‐year time series of monthly to bimonthly samples of V6–V8 amplicons of the 16S rRNA gene for the entire bacterial assemblage. While the 16S rRNA gene is generally not preferred for clade designation (Mazard *et al*., 2012), clade assignment within regions of this gene is possible (Post *et al*., 2011; Mackey *et al*., 2017). We characterize the relative abundance of different *Synechococcus* oligotypes through time and analyse patterns with compositional data analysis techniques. It is increasingly recognized that high‐throughput sequence data are compositional in nature (Gloor *et al*., 2016; Egozcue *et al*., 2020), and that analysis of this data type requires appropriate tools that take into consideration the distinct challenges of data belonging to a constrained subset of real space (Aitchison, 1986). Common methods of analysis for sequence data, if they do not account for the sample space, can lead to misleading interpretations and errors (Gloor *et al*., 2016; Chong and Spencer, 2018). With this approach, we are able to find direct links between changes in *Synechococcus* composition and different environmental variables. We also provide insight into how the underlying diversity structure shapes *Synechococcus* abundance features at MVCO. Together, these findings contribute insight into mechanisms that help resolve the paradox of diversity within this important marine cyanobacteria.

## Results

### *Synechococcus* mock communities

We constructed two mock communities to help identify biases in our extraction, amplification and sequencing pipeline. Mock communities were comprised of equal concentrations of six or seven different *Synechococcus* strains previously isolated from MVCO (Table S1). For communities 1 and 2, 96.1% and 95.5% respectively, of the taxonomically labelled *Synechococcus* sequences were able to be grouped into an oligotype with the parameters we chose, and we recovered all the *Synechococcus* strains that comprised each community. The representative sequence of each oligotype was an exact match to the strain reference 16S sequence. We note that less restrictive parameter values for oligotyping would result in additional oligotypes, with total amounts of a few hundred sequences. This observation allows us to discern what can be reliably labelled as true sequence diversity versus sequencing noise (with the caveat that we expect no native deviations or subpopulations of 16S genotypes within our *Synechococcus* cultures).

Replicate runs differed in the amount of total (and thus *Synechococcus* sequences) generated (Table S2), but the proportions of each strain appeared consistent across replicates (Fig. S1). These proportions, however, deviated from an equal distribution among strains from 0.045 to 0.345 for community 1 (expected = 0.143) and 0.051 to 0.292 for community 2 (expected = 0.166), with the assumption that each strain here would have two copies of the 16S rRNA gene as common for most clades (Fuller *et al*., 2003; Ahlgren and Rocap, 2012). It is unknown if these deviations are from varying copy number of the 16S rRNA gene, amplification bias, cell physiological state, or possible differences in ploidy level (Perez‐Sepulveda *et al*., 2018). As the communities used different strain mixes, it is difficult to identify any strain‐specific trend toward over or under representation. However, strains belonging to clade I tended to be under‐represented.

### *Synechococcus* at MVCO

**Fig 1:**
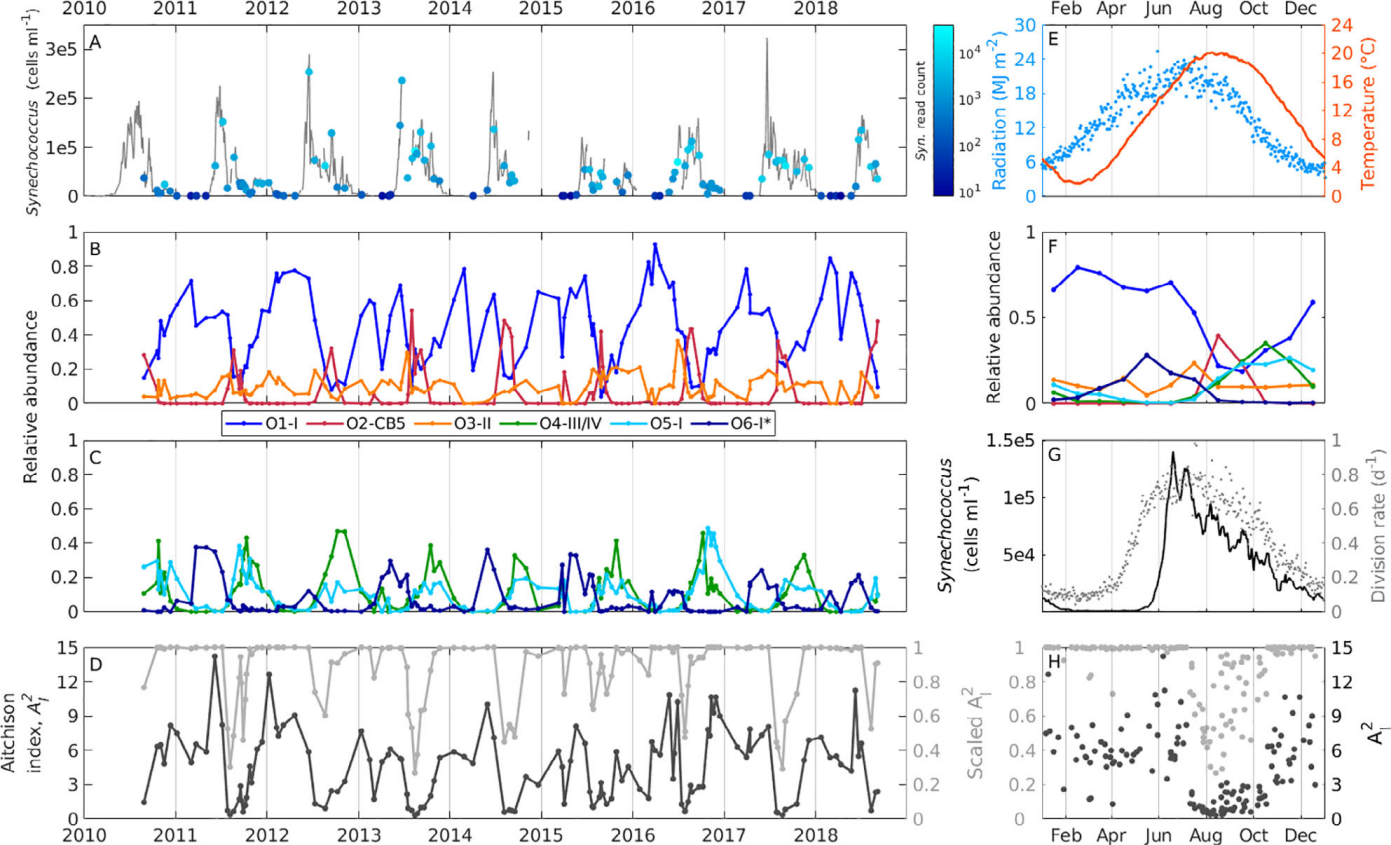
A. MVCO time series of *Synechococcus* (grey line, from flow cytometry) and sample time points for amplicon data. Colour indicates total *Synechococcus* sequence reads (log scale). Time series of relative abundance of *Synechococcus* oligotypes (B) O1‐I, O2‐CB5 and O3‐XV and (C) O4‐III/IV, O5‐I and O6‐I*. Relative abundance is oligotype sequence read count divided by total *Synechococcus* reads per sample. Colour indicates oligotype as in legend. D. Aitchison index $A_{I}^{2}$ (black line) and scaled $A_{I}^{2}$ (grey line) calculated from Eq. 11 from six most abundant oligotypes. E. Year day climatology (average value across year days) at MVCO of incident radiation (light blue dots) and temperature (orange line). F. Center of oligotype relative abundances calculated with Eq. 13 of zero‐imputed samples belonging to each month for six most abundant oligotypes. Colour indicates oligotype as in (B) and (C). G. Year day climatology of *Synechococcus* concentration (black line) and population division rate (grey dots). H. Plot of $A_{I}^{2}$ and scaled $A_{I}^{2}$ over year day, colours same as D. [Color figure can be viewed at wileyonlinelibrary.com]

A total of 12 540 274 sequences were merged from the environmental time‐series samples. Of these, 319 270 were identified as *Synechococcus*. The percentage of *Synechococcus* reads relative to total reads varied from 0.005% to 17.5% per sample, with a median value of 1.5%. The percentage of *Synechococcus* reads tended to track with flow‐cytometry‐derived *Synechococcus* cell concentration (Figs 1A and Fig. S2), such that the highest proportions were observed when cell concentration was >∼10^5^ cells ml^−1^, and very few *Synechococcus* reads were found when cell concentration was a few hundred cells ml^−1^.

Resulting oligotypes had a purity score of greater than 95, and 96% of sequences were able to be grouped into 14 oligotypes with the parameters we chose. Six main oligotypes accounted for 89% of total *Synechococcus* sequence reads. From our custom database of *Synechococcus* full‐length 16S sequences, we were able to find a direct match to single or multiple clades for most, but not all, of the 14 oligotype representative sequences (Table S3; Fig. S3). Throughout the text, we refer to oligotypes with an ‘O’ followed by a number representing rank order for number of sequence reads followed by the best clade match. For some oligotypes, no direct match to any cultured isolate was found, but closest matches were typically only one base pair different. The exception was O6‐I*, which was three base pairs away from the closest match to clade I strains (here ‘*’ denotes the uncertainty in this oligotype match). Oligotype O4 matched both to clades III and IV, which share identical V6–V8 sequences. Oligotype O4‐III/IV could belong to clade III or IV, and it is not clear if this oligotype represents one or both of these clades. Representatives of each clade have been isolated at MVCO [(Hunter‐Cevera *et al*., 2016b) and SI].

Both O1‐I and O5‐I matched strains of clade I, but we found that these oligotypes tended to match strains that partitioned into different subclades of clade I. O1‐I matched strains that belong to subclade IC, while O5‐I matched those of subclade IE from *ntcA* designations following Hunter‐Cevera *et al*. (2016b). Subclade IC appears to be grouped with subclade Ib as described with the *petB* marker (Mazard *et al*., 2012) for reference. Type O6‐I* had no cultured representative in our database, but we believe this oligotype likely represents another subclade division within clade I. At least four different subclades were previously detected at MVCO (Hunter‐Cevera *et al*., 2016b), but only two have cultured representatives (IC and IE).

As with the mock communities, we found consistent proportions among the *Synechococcus* oligotypes across samples that were processed two or three times (separate amplifications and sequencing runs, see Fig. S4). Only when the total number of *Synechococcus* reads dropped below ∼15 we did observe large differences in the composition, with stochastic presence or absence of oligotypes. As described in the methods, later in the time series, seawater was filtered onto PES disk filters rather than Sterivex cartridges. For the available samples for which both Sterivex cartridges and disk filters were processed, we found almost no difference between *Synechococcus* proportions for the mock community (Fig. S1B) or for sample Sept‐5‐2018 (Fig. S4) between Sterivex and PES filter samples.

#### Seasonal patterns

**Fig 2:**
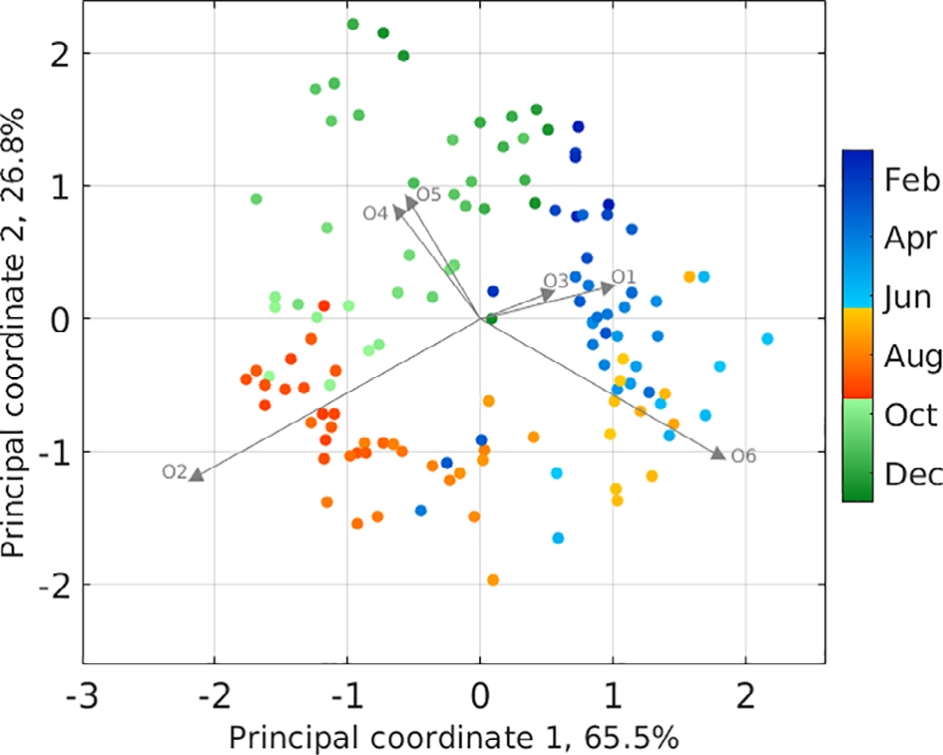
Covariance biplot of clr‐transformed, centred, zero‐imputed data. Rays represent six oligotypes and have been scaled by $1/\sqrt{\left(n-1\right)}=1/\sqrt{128}$ to bring values onto scale of log‐ratio variance and covariance. Sample projections are represented by filled circles and have been scaled by 128 to be visible on plot. Colour indicates sample year day. [Color figure can be viewed at wileyonlinelibrary.com]

Of the 14 oligotypes, 10 displayed highly consistent, repeatable annual patterns of relative abundance (Fig. 1B,C,F, Fig. S5). This seasonality can be readily observed in a biplot of the data and how projections of sample data appear as a circular pattern over corresponding oligotype vectors (Fig. 2). Strong similarity among compositions within each season was also found by calculating the Aitchison distance (a measure of dissimilarity, see Experimental procedures) pairwise between each sample composition (Fig. S6).

The most relatively abundant oligotype, O1‐I, dominated *Synechococcus* sequences in winter through end of spring, comprising more than 50% of the reads during these months. In spring, O6‐I* comprises up to 25% of the reads, but otherwise remains at a relatively low percentage of the population for the rest of the year. All other oligotypes are either not present or in low relative abundance during this time. This unevenness in the composition is reflected in the Aitchison index [$A_{I}^{2}$, a measure of evenness across composition classes (Egozcue and Pawlowsky‐Glahn, 2019), see Experimental procedures, Fig. 1D and H]. Large values of $A_{I}^{2}$ (or values close to 1 for scaled $A_{I}^{2}$) indicate that only one or two classes dominate a composition.

Late summer to early fall appeared to be the most diverse time (with regard to evenness) as indicated by low values of $A_{I}^{2}$. The second most abundant oligotype, O2‐CB5, had a very defined relative abundance peak during this time, and was usually not detected outside of this summer period. In summer, O1‐I decreased in relative abundance, while O4‐III/IV and O5‐I* began to increase. These two oligotypes peaked in early fall at around 20%–30% of *Synechococcus* sequence reads.

Oligotypes O4‐III/IV and O5‐I followed very similar seasonal relative abundance patterns. This is reflected in a low Aitchison variation value (Table 1), indicating that the ratio between these oligotypes is fairly constant. The covariance structure between oligotypes (and relationship to individual samples) can also be observed within a biplot. The interpretation of a biplot of compositional data is not necessarily the same as for unconstrained data, and the reader is referred to Aitchison and Greenacre (2002) for more information. The distance between ray end points (i.e. links) represents the variation between the corresponding log ratio. The relatively short links between O4–O5 and O1–O3 (almost coincident vertices) indicate that these ratios are rather constant (Fig. 2).

**Table 1:** Values of Aitchison variation calculated from Eq. 15 for compositions constructed of the six most abundant oligotypes, zero imputed.

	O2	O3	O4	O5	O6
O1	12.74	0.84	4.33	3.48	3.18
O2		9.98	7.21	7.6	16.66
O3			2.58	2.63	3.89
O4				1.03	10.24
O5					10.16

Smaller values indicate higher proportionality among components.

The longer links between O2 or O6 and other oligotypes (Fig. 2) indicate higher variation of those ratios, which is also indicated by higher Aitchison variation values (Table 1). In particular, the Aitchison variations between O2 and other oligotypes stand out, indicating little or no proportionality with other types. This is consistent with the rapid appearance of O2 in the summer, when other types show low relative abundance. The shorter ray of O3‐XV indicates a low variability of the clr‐transformed component. O3‐XV had a consistently low relative abundance over the annual cycle, typically hovering at less than 20% of the *Synechococcus* reads, and only reaching a relatively small maxima in mid‐summer.

Other lower‐abundance oligotypes also displayed distinct seasonal patterns (Fig. S5). Types O7‐IX and O8‐CB5 displayed a peak in relative abundance in summer, similar to O2‐CB5. We found four other oligotypes that appeared to belong to clade I, (O9, O10, O11 and O13), but these did not display any consistent seasonal pattern. We cannot resolve if these types represent sequencing error or true diversity within clade I. Oligotypes were also identified belonging to clade VI, O12, and clade II, O14. These types appeared only in summer, and both oligotypes were a direct match to strains isolated from MVCO (Table S3).

#### Relationships with environmental variables

**Fig 3:**
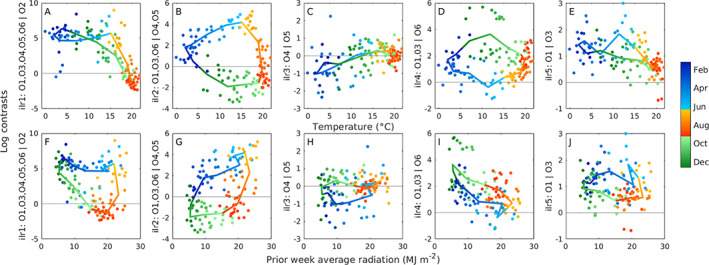
Relationship between log contrasts (ilr coordinates from ilr transformation) and temperature (A–E) and average weekly radiation prior to sampling (F–J). Colour indicates year day and season. Monthly climatological relationships are indicated by colour line (average values within each month). The zero line is indicated in each plot for reference. The *y*‐axis in each panel provides information about the relative importance of oligotypes (or groups of oligotypes) in relationship to each other. [Color figure can be viewed at wileyonlinelibrary.com]

We focus our remaining analysis on the six most abundant oligotypes, which comprise 89% of the *Synechococcus* reads. While other oligotypes demonstrate seasonality, a low number of sequence reads for the majority of the year precludes a thorough seasonal analysis. To relate changes in *Synechococcus* composition to available environmental variables, we utilize the isometric log ratio (ilr) transformation (Egozcue *et al*., 2003; Pawlowsky‐Glahn *et al*., 2015). The transformed data are real, unbounded values, enabling the use of familiar statistical approaches. This transformation results in weighted log‐contrasts of oligotype proportions that have been grouped to provide informative comparisons and capture all the variability within the subcomposition of these six oligotypes (see Experimental procedures, Fig. S7; Table S4). Contrasts are interpreted as the relative contribution of oligotypes (or groups of oligotypes) in relationship to each other. For example, the first contrast separates the contribution of O2 from the rest of the composition (Fig. 3A and F), while the second (Fig. 3B and G) compares oligotypes that are relatively more abundant in spring (O1, O3, O6) with those that are more abundant in the fall (O4, O5). Subsequent contrasts explore comparisons within each of these groupings.

**Fig 4:**
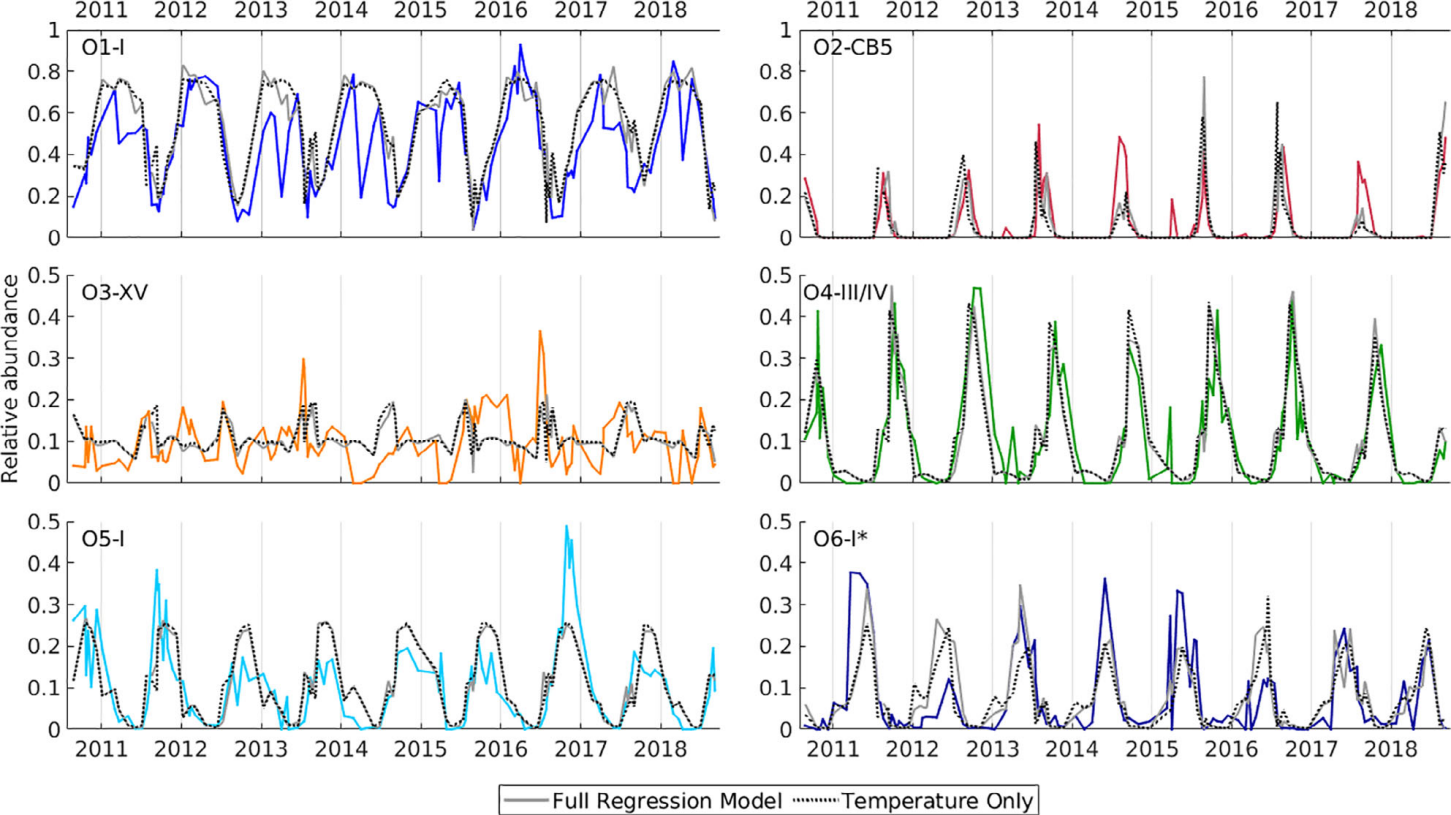
Time series of relative abundance of six most abundant oligotypes (colour line in each plot, as in Fig. 1B and C), with modelled compositions from best fit multivariate regression parameters of the full model (solid grey line) and temperature‐only model (dashed black line). [Color figure can be viewed at wileyonlinelibrary.com]

The ilr transformation with standard multivariate regression within different seasons allows us to identify links between environmental variables and compositional changes (Fig. 3, Fig. S8, S9, S10). We delineate seasons based on *Synechococcus* population dynamics (Hunter‐Cevera *et al*., 2020a), but for which we also observe differences in composition and log contrasts for each season (Fig. S8). We find that the seasonal change in diversity can be well explained solely from ‘bottom‐up’ factors. Temperature and weekly averaged incident solar radiation explained significant variability in all seasons, and phosphate concentration was found to be significant in summer (Table 2). Silicate and ammonium had nearly significant *p*‐values in different seasons (winter/spring and summer for silicate, fall for ammonium, see Table S5). Seasonal fitting with significant variables allowed us to reproduce observed relative abundance patterns of each oligotype (Fig. 4). Notably, a regression model using temperature alone reproduced qualitative features well, highlighting the importance of this variable.

**Table 2:** Variables identified as significant per season in multivariate regression with ilr coordinates.

Season	Variable	Λ	*p*‐value	O1‐I	O2‐CB5	O3‐XV	O4‐III/IV	O5‐I	O6‐I*
Winter/spring *n* = 43	Temperature	0.321	2.85 × 10^−8^	0.185	0.155	0.173	0.157	0.145	0.185
	Weekly averaged light	0.739	0.0406	0.159	0.167	0.165	0.163	0.159	0.185
	Temperature	0.277	5.84 × 10^−10^	0.091	0.336	0.113	0.193	0.192	0.076
Summer *n* = 46	Phosphate	0.431	2.44 × 10^−6^	0.004	0.876	0.007	0.04	0.074	0.0001
	Weekly averaged light	0.658	5.61 × 10^−3^	0.187	0.137	0.182	0.156	0.151	0.187
Fall *n* = 40	Temperature	0.148	3.82 × 10^−13^	0.137	0.179	0.155	0.182	0.152	0.195
	Weekly averaged light	0.726	0.045	0.162	0.23	0.155	0.149	0.162	0.142

Wilk's Λ and *p*‐values are given for each variable, and each row refers to added significance of that variable compared to model constructed of variables listed in the above rows within each season. For first row of each season, Λ and *p*‐values refer to full model, whereas values in subsequent rows refer to the significance of only one added variable. *p*‐Values are calculated from F‐distribution approximation. O1–O6 columns list slope parameters from best multivariate fit that have been back‐transformed with the ilr inverse calculation (Eq. 32). These values are interpreted as the perturbation applied to a composition for one unit increase of corresponding variable.

In addition to enabling multivariate analysis, the ilr transformation also provides comparative information about the groups that comprise each log contrast. This enables insights into possible environmental preferences of oligotypes. We also find insight into oligotype environmental responses from the transformation of regression model slope parameters back to the simplex. Parameter compositions are interpreted as the perturbation applied to a composition if the variable increases by one unit (Van den Boogaart and Tolosana‐Delgado, 2013). These differ for each oligotype and in each season, with larger values indicating a larger response to an increasing variable (Table 2).

## Discussion

We find that the *Synechococcus* population at MVCO is comprised of 14 different oligotypes (linked to various clades and subclades), and that 10 of the 14 oligotypes demonstrate a remarkably consistent seasonal pattern of relative abundance (Figs 1 and 2, Fig. S5). The regularity of these patterns suggests that strong drivers, environmental or biological (or both), govern *Synechococcus* microdiversity dynamics at this temperate location. Our measurements, however, are proportions, and this data type poses distinct challenges for analysis. The data are interdependent due to the fixed limits of the number of sequences that can be generated on sequencing platforms and should be thought of as a random sample of relative abundance (Gloor *et al*., 2016). In addition, direct interpretation of proportions can be problematic as these are influenced by gene copy number, physiological cell state, amplification bias and abundance of other organisms in the sample. Results from our own mock communities suggest that sequenced proportions here may not reflect precise proportions of cell types in the field. Other researchers have also found biases in final sequence proportions of mock communities or mixed DNA samples (Salipante *et al*., 2014; Schirmer *et al*., 2015; Ahlgren *et al*., 2019).

These analysis challenges can be addressed with the use of compositional data analysis techniques (Aitchison, 1986; Pawlowsky‐Glahn *et al*., 2015; Gloor *et al*., 2016). Intrinsic in this approach is the realization that meaningful information lies in the ratio of proportions, rather than in the absolute value of the proportions themselves (Aitchison, 1986; Pawlowsky‐Glahn *et al*., 2015). Compositional data analysis focuses on how proportions change relative to each other, enabling insight into the drivers of compositional change despite the limitations and biases listed above. This type of analysis is also *subcompositionally coherent*; analysis of a subset of the data yields the same result as if all the data had been considered, preventing errors that arise from inclusion or exclusion of different taxa. Different results based on choice of normalization with different denominators (such as those encountered in Larkin *et al*., 2020) are also avoided. Here, we utilized these techniques to not only address these data type challenges but also to directly relate changes in *Synechococcus* compositions to environmental variables.

With the ilr transformation and multivariate regression, we identified significant seasonal responses to temperature and light, along with phosphate. The effects of temperature and light on diversity dynamics are consistent with the strong roles these factors play in *Synechococcus* population dynamics (Hunter‐Cevera *et al*., 2020a). We utilize our current understanding of the *Synechococcus* population at MVCO to help interpret observed diversity patterns and their relationships to environmental variables.

Winter is a particularly challenging season for *Synechococcus* at MVCO. Cold winter temperatures (0–5 °C) severely limit cell division and cell concentration rapidly declines in this season. We observe an equally dramatic decrease in oligotype diversity (Fig. 1D and H), suggesting that winter is challenging for most *Synechococcus* clades at this location. The proportion of oligotypes dwindle in the winter until the population is dominated by just O1‐I (>80%), suggesting a better tolerance of cold conditions for this oligotype. The concentration of cells in winter depends on how long and the extent to which temperature remains below 5–6 °C, the threshold above which we observe a significant increase in division rate (Fig. 1E and G, (Hunter‐Cevera *et al*., 2020a)). As that threshold is crossed, a spring bloom is triggered. The bloom is initially comprised of oligotype O1‐I. We believe that successful overwintering coupled with the apparent ability to divide at low temperatures enables the dominance of O1‐I early in the bloom.

As spring warming continues, the bloom advances – cell concentration increases by 2–3 orders of magnitude over the span of a few months – and competitors with O1‐I begin to appear. First another clade I type, O6‐I*, increases in relative abundance in late spring. These findings are consistent with knowledge of clade I physiology and biogeography. Clade I is typically found in cold, mesotrophic coastal waters (Zwirglmaier *et al*., 2007; Huang *et al*., 2012; Sohm *et al*., 2016), and has even been observed in Arctic regions (Paulsen *et al*., 2016). Clade I strains can divide faster than other clades at low temperatures and they can better tolerate cold shock (Pittera *et al*., 2014). This cold tolerance is attributed to increased stability of light‐harvesting complexes and likely better membrane fluidity at cold temperatures (Pittera *et al*., 2017, 2018).

While low temperatures favour clade I, warming waters at the end of spring and in summer are associated with the sequential appearance of other clades. Oligotypes follow a remarkably consistent cyclic succession pattern of relative abundance (Figs 1B,C,F and 2). In mid‐summer, O3‐XV shows a small increase in relative abundance and may prefer warmer conditions (Fig. 3E). The third most abundant type belongs to clade XV. We note that Mazard *et al*. (2012) incorporate clade XV as a subclade of clade II (subclade h), but we keep a clade XV designation here for continuity with previous literature. Clade XV has been detected in low abundance in transitional waters between distinct ocean biomes (Sohm *et al*., 2016). The low relative abundance at MVCO is consistent with low detection in these other oceanic regions. Clade XV was observed by Farrant *et al*. (2016) (detected as a clade IIh) only in cooler water (14.1–17.5 °C) across global samples. To our knowledge, our observations here are the first detection of clade XV in colder coastal waters, and it is possible that O3‐XV represents a subclade (or subclades) of clade XV/II that is better adapted to the relatively colder conditions at MVCO.

By late summer, we observe a dramatic increase in O2‐CB5 contribution, until it dominates the *Synechococcus* sequences (>40%). By early fall, O2‐CB5 has all but disappeared from the sequence data. Relationships between temperature and log contrasts (ilr coordinates) indicate that this pattern may be due to a temperature response. Oligotype O2‐CB5 typically only increases in relative abundance after water temperature exceeds 13–15°C (as seen in Fig. 3A by the decrease in ilr_1_ with temperatures above this range). O2‐CB5 also shows a strong response to phosphate as indicated by regression slope parameter values (Table 2). However, since this response is per unit for a given variable, and we rarely observe >1 μM levels of phosphate at MVCO, this effect does not translate into a large effect on fitted compositions (Fig. 4). To the best of our knowledge, the physiology of clade CB5 has not been studied; it will be important to characterize the physiology of this clade to better understand and interpret our observations here.

In the temperate water at MVCO, temperatures continue to rise until the end of summer. The transition to a fall composition begins when water temperature reaches ∼16–18°C. Illustrated by the log contrast between spring and fall types (Fig. 3B), the shift toward O4‐III/IV and O5‐I becomes apparent at this temperature. These two oligotypes eventually comprise ∼20%–40% of the *Synechococcus* sequences in fall. Little is known about the temperature dependence of clades III and IV. Physiological studies have shown that representatives of the subclade to which O5‐I belongs have maximal division rates at a higher temperature than representatives of the subclade of O1‐I (Pittera *et al*., 2014), which may help explain the prevalence of O5‐I later in the season. (Note that within clade I, O5‐I is grouped in a separate subclade from O1‐I and O6‐I* [see SI, (Hunter‐Cevera *et al*., 2016b)].

In fall, temperatures begin to decline as does light, and division rate is primarily limited by light in this season (Hunter‐Cevera *et al*., 2020a). This limitation results not only from the seasonal decline in light level but also significant attenuation by an increase in eukaryotic phytoplankton [Sosik unpublished data, (Hunter‐Cevera *et al*., 2020a)]. We hypothesize that O4‐III/IV and O5‐I are better adapted to very low light conditions than other types. These two oligotypes share very similar seasonal relative abundance patterns, despite belonging to different clades. This similarity may be an example of convergent evolution wherein genetically separate clades find similar solutions to environmental challenges (Sohm *et al*., 2016). While very similar, examination of the third log contrast, which compares these two oligotypes, indicates that O4‐III/IV may be favoured in slightly warmer conditions over O5‐I (Fig. 3C).

In addition to seasonal patterns of *Synechococcus* cell concentration at MVCO, there are notable subseasonal variations, with changes of up to an order of magnitude over days to weeks (Fig. 1A). We previously suggested these shorter timescale abundance changes might be due to different clade types increasing or decreasing in succession (Hunter‐Cevera *et al*., 2020a). To first order, the data presented here are not consistent with this idea; oligotype clade patterns shift on the seasonal timescale, rather than at finer scales. It is possible that changes may be occurring at even finer taxonomic resolution, such as those observed by Ahlgren *et al*. (2019) for amplicon sequence variants (ASVs) off the coast of California, where variations in ASVs within clades were correlated with viral community structure. Biological factors, such as protist grazing (Zwirglmaier *et al*., 2009; Apple *et al*., 2011) or viral predation (Mann, 2003; Mühling *et al*., 2005), can be clade‐specific and could contribute to the variation that is not explained by environmental factors at MVCO. Activities and interactions with other abundant cells, such as eukaryotic phytoplankton or heterotrophic bacteria, could also directly or indirectly affect *Synechococcus* dynamics (Ramanan *et al*., 2016). These factors would be especially important to consider for O3‐XV, whose variability is not well captured within our regression model (Fig. 4). The short time scale variations in abundance at MVCO are consistent with predator–prey type oscillations (Hunter‐Cevera *et al*., 2020a), but analysis of the time series with a higher resolution genetic marker would be required to determine whether these abundance oscillations coincide with finer‐resolution sequence composition changes.

A discussion of the influence of bottom‐up factors on diversity would not be complete without consideration of nutrients, which are critical for cell growth. To first order, we find that, for *Synechococcus*, nutrients are not among the main factors governing clade composition at MVCO. Only phosphate was found to explain significant variability within the *Synechococcus* compositions and only during summer. We note though that both silicate and ammonium were found to have nearly significant *p*‐values (Table S5), and their importance may emerge with longer or higher‐frequency time‐series sampling. Silicate is particularly interesting, given the recent observations that *Synechococcus* can accumulate this element (Baines *et al*., 2012).

While we have identified temperature and light as important abiotic variables that affect *Synechococcus* diversity, it is the time scale of changes of these variables that critically shape the composition. Hutchinson (1961) proposed temporal environmental variability as a potential resolution to the paradox of the plankton. Environmental variability also appears to explain the persistence of microdiversity in *Synechococcus* at MVCO. Our observations and analysis of the striking cyclic diversity dynamics suggest that oligotypes have distinct light and temperature preferences. These different preferences coupled to seasonal environmental changes enable each type to persist but not to dominate the assemblage over an entire year. In particular, differential seasonal temperature responses have enabled us to well reproduce oligotype relative abundance patterns solely through a multivariate linear regression model. Intrinsic in this approach is the allowance for seasonal differences in temperature response, which would be expected for oligotypes that have different temperature preferences and growth optima.

To persist, oligotypes must also be able to withstand temporary unfavourable conditions. Cold wintertime temperatures are challenging for all *Synechococcus* oligotypes at MVCO (Fig. 1A). The relative ability of O1‐I to survive in low temperatures appears to enable its dominance in winter and spring. It is not clear if other oligotypes also successfully overwinter at MVCO or if they are resupplied from warmer shelf waters and then thrive when conditions are favourable.

Our results also highlight the importance of light and especially temperature as physiological avenues for differentiation among picocyanobacteria. Stark differences in responses to these two variables can even be found within a single clade. We observe three different clade I oligotypes that appear to differ in their responses to these variables. Differentiation among such closely related members offers a case study for both the drivers and constraints that determine diversification.

Our findings also underscore the importance of understanding the diversity structure within a population to fully understand abundance dynamics. For example, O1‐I dominates the assemblage in winter and spring, such that the spring bloom dynamics are determined largely by the physiology and ecology of this oligotype. In contrast, abundance dynamics in summer and fall are a composite of multiple oligotypes. How different types contribute to overarching population features is especially critical to understand if we are to predict how populations will shift in response to future climate change. Increases in water temperature could have profound impacts on *Synechococcus* diversity at this location; warmer winters could allow increased abundance or survival of different oligotypes, and warmer spring and summer temperatures would enable longer periods of growth for oligotypes that prefer warmer conditions. How diversity shifts translate to abundance features would depend on the distinct growth and loss processes of each type. It will be important to explore the ecophysiological attributes of each oligotype to better understand the links between diversity, large‐scale abundance patterns and related ecosystem processes.

Resolution to many of these questions will also require higher frequency sampling, coupled with techniques that enable actual cell counts of different *Synechococcus* types. Automated measurement and sampling platforms that enable storage of samples for later analysis is an exciting area of development (Yamahara *et al*., 2019; Hansen *et al*., 2020). Flow cytometry and development of microfluidic platforms, in particular, have the potential to be able to monitor different cell populations when combined with fluorescence *in situ* hybridization (Huber *et al*., 2018). Continued development of these approaches combined with automation will provide the necessary tools to be able to monitor, measure and ultimately better understand the diversity and dynamics of ocean microbes.

## Experimental procedures

### Sample collection and DNA extraction

As part of the on‐going Northeast U.S. Shelf Long Term Ecological Research (NES‐LTER), seawater samples were collected near the MVCO offshore tower (41°19.500′ N, 70°34.0′ W) at roughly bimonthly to monthly intervals over an 8‐year period from August 2010 to October 2018 for a total of 129 samples. Water was sampled at the surface via bucket sample or at 2 m depth with Niskin bottles attached to a rosette sampler on board the R/V Tioga. Two to three litres of surface seawater were pre‐filtered through a 20 μm Nitex® mesh and then filtered onto 0.2 μm Sterivex® cartridge filters (Millipore) under vacuum pressure of no more than 40 kPa for samples up until fall of 2017. After this, samples were filtered onto Sterivex cartridges via a peristaltic pump (MasterFlex) at the lowest speed of ‘1’ up until Summer 2018. After this time, samples were no longer pre‐screened at 20 μm and were filtered onto 47 mm PES 0.2 μm disk filters (Millipore) with vacuum filtration. The last sample in this time series was filtered onto both a Sterivex cartridge and PES disk filter for comparison. Samples were frozen at −80°C dry or with cell lysis buffer.

For DNA extraction, samples were thawed on ice. Disk filters were cut into smaller pieces with sterile scissors. Approximately 200 μl of autoclaved 0.5 mm zirconia‐silica beads (BioSpec Products) were added to the cartridges or disk filters. Cell lysis buffer was added if sample had been frozen dry. Samples were shaken vigorously at 2500 rpm for 10 min on a benchtop vortexer. DNA extraction then followed a modified procedure with Qiagen Puregene kit reagents as described in Palacios *et al*. (2008). DNA concentration and purity were determined with a NanoDrop 2000 spectrophotometer (ThermoScientific) as almost all samples yielded a concentration of ≥30 ng μl^−1^.

Water temperature, light measurements, nutrient concentrations and *Synechococcus* cell concentration from automated flow cytometry and division rate at MVCO for this time series can be found at Hunter‐Cevera *et al*. (2020b), with methods as described in Hunter‐Cevera *et al*. (2020a).

### Mock *Synechococcus* communities

To identify potential biases in the DNA extraction, amplification and sequencing pipeline, we constructed mixed mock communities of non‐axenic *Synechococcus* strains. Two communities were constructed with different strains (Table S1) and processed slightly differently from each other. For community 1, cell concentration of late exponential phase culture of each isolate was measured with a Guava Easy Cite flow cytometer. Aliquots of each culture were added to two individual 2 L flasks of filtered MVCO seawater to a final concentration of 1.25 × 10^4^ cells ml^−1^ per strain. Only duplicate samples were constructed for this mock community. Each 2 L volume containing the strain mixture was vacuumed filtered onto Sterivex filter cartridges.

For community 2, cell concentration of each strain was measured with a FACSCalibur flow cytometer connected to a syringe pump as described in Hunter‐Cevera *et al*. (2014). Each strain was added to a common carboy of 8 L filtered MVCO seawater to a final concentration of 10^4^ cells ml^−1^ per strain. From this carboy, triplicate filters were prepared by filtering approximately 2 L onto a Sterivex filter cartridge via peristaltic pump. The remaining 2 L was filtered onto a PES disk filter with vacuum filtration. Filter processing, DNA extraction, PCR amplification and sequencing were the same as described for environmental samples.

### Amplification and sequencing of V6–V8 region

The hypervariable V6–V8 region of the 16S rRNA gene (ca. 464 bp) was PCR amplified with general primers 926F (5′‐AAA‐CTYA‐AAK‐GAA‐TTG‐ACG‐G‐3′) and 1392R (5′‐ACG‐GGC‐GGT‐GTG‐TRC‐3′) that were extended with sequences and required adapters and barcode or index regions for Illumina sequencing. Total primer length was 79 or 83 base pairs (IDT). Reactions contained AmpliTaq Gold 360 Master Mix (Applied Biosystems), 0.2 μM forward and reverse primers, 15 ng of DNA template and water (Ambion) in a total 32 μl volume. Cycling conditions were 95°C for 3 min; followed by 30 cycles of 30 s at 95°C, 45 s at 55°C, and 1 min at 72°C; with a final extension step of 72°C for 5 min. Presence of positive products was checked by gel electrophoresis. For each sample, triplicate reactions were performed and subsequently pooled. Control samples of water to check for contamination were run for every unique pair of barcoded and indexed primers. Product cleaning, quality control and sequencing on the MiSeq (Illumina) were performed at the Marine Biological Laboratory Keck Sequencing Facility (Woods Hole, Massachusetts) according to their protocols. Environmental samples were sequenced over six different MiSeq runs, beginning in 2016 and ending in 2019. Multiple runs allowed some environmental samples to be amplified and sequenced two or three times, and sequence data were pooled for these samples.

### Sequence taxonomy and *Synechococcus* clade identification

Reads were demultiplexed based on the combination of index and barcode with custom bash scripts from the Keck Facility. Primers were removed and corresponding reads merged with the package illumina‐utils (github.com/merenlab/illumina-utils). Only reads with three or less mismatches in the merged region and a quality score of greater than Q30 for two‐thirds of the unmerged region (‘Q30 check’) were kept. Taxonomy was assigned to reads within the VAMPs pipeline (Huse *et al*., 2014), using the Global Assignment of Sequence Taxonomy (GAST) with RefSSU, a primary reference database of near full‐length reference sequences, derived from the SILVA rRNA database project (version 119).

In addition to GAST taxonomy assignment, we also screened sequences with a custom database of roughly full‐length 16S rRNA sequences of *Synechococcus* isolates (Table S6). This database contained a total of 191 *Synechococcus* sequences for which unambiguous clade designation was available based on a separate, higher resolution diversity marker (i.e. 16S ITS, *ntcA*, *petB*). This database included 16S sequences from strains isolated from MVCO (see SI). All unique environmental sequences were checked for similarity against this database with blastn (v. 2.9.0). Sequences that had a bitscore of >700 against this database were included. For both the environmental samples and mock communities, all sequences that were identified as *Synechococcus* by GAST met these criteria, but a small number of sequences (a few hundred) not identified as *Synechococcus* by GAST were also included (most notably sequences labelled as ‘Cyanobium’ for the mock communities or those identified only to the order ‘Chroococcales’ for environmental samples).

#### Oligotyping and clade assignment

We performed oligotyping (Eren *et al*., 2013) to identify meaningful variation and reduce impact of sequencing noise. Sequences identified as *Synechococcus* were aligned with PyNAST (v 0.1) against a Green Genes database reference alignment (v. 6Oct2010, greengenes.lbl.gov). Uninformative gap regions were removed via script from oligotyping package (Eren *et al*., 2013). With this package, aligned environmental sequences were grouped into distinct sequence types (oligotypes).

Sequences from the mock communities were aligned and oligotyped separately from the environmental sequences and from each other. For mock communities, oligotypes were formed with parameters *A* = 400 (minimum total abundance of an oligotype) and *c* = 13 (number of positions to use for constructing oligotypes, selected from nucleotide positions with highest entropy). These parameters were selected for the minimum number of positions and abundance that would allow the recovery of only six or seven oligotypes, which should comprise communities 1 and 2 respectively. For environmental samples, oligotypes were selected with parameters *M* = 200 (minimum substantive abundance) and *c* = 19. These parameters were chosen based on mock communities, as we expected environmental samples to be more diverse with potentially lower abundances of oligotypes.

Clade matches for *Synechococcus* oligotypes of both environmental and mock communities were found via alignment of representative oligotype sequences to the V6–V8 region of the *Synechococcus* reference database (Table S6) with the BioAlignment package (v1.0.1) in Julia (v 1.2.0). Unique V6–V8 sequences by clade in the database were also identified via alignment. Secondary unique sequences within each oligotype that were relatively abundant (greater than 50 reads) were further screened to ensure that the closest *Synechococcus* clade match was the same as the representative oligotype sequence.

### Compositional data analysis

We follow standard compositional data analysis techniques and provide additional information and an overview below for readers who are unfamiliar with this type of analysis. The reader is referred to Aitchison (1986) and Pawlowsky‐Glahn *et al*. (2015) for in‐depth background.

Compositional data are data that are parts of a whole (e.g. fractions), and as such are subject to a unit sum constraint

(1) $$x_{1}+x_{2}+\cdots +x_{D}=1,$$

where *x*_*j*_ ≥ 0 is an individual component of a composition of *D* parts. Because of this constraint, the components of the composition are not independent. The intrinsic dependency between components poses challenges for analysis. In particular, the associated sample space of compositional data is not ${\mathbb{R}}^{D}$, but rather the simplex, $S^{D}$, the set of all possible compositions satisfying the constraint (1):

(2) $$S^{D}=\left\{x=\left[x_{1},x_{2},\ldots x_{D}\right]:x_{j}\geq 0,\sum_{j=1}^{D}x_{j}=1\right\}.$$

The methods of compositional data analysis appropriately account for this geometry with operations specific to the simplex or with transformations that enable analysis in the more familiar real space. The transformations typically involve log ratios, as the meaningful information in relative data is found in the ratio of proportions to one another and how they vary (Aitchison, 1986).

Our environmental samples are partitioned into 15 different ‘groupings’ of *Synechococcus*: 14 oligotypes (representing either subclades, clades or grouping of clades of *Synechococcus*) and a 15th category of *Synechococcus* sequences that we were unable to group into an oligotype. We focus our analysis on the relative abundance patterns of only the six most abundant *Synechococcus* oligotypes, which comprise ∼89% of total *Synechococcus* reads across the environmental samples. For each sample *i*, we used the number of counts of oligotype *j* (call these counts *c*_*ij*_) to form the subcomposition **x**_*i*_, a 1 × *D* row vector whose elements *x*_*ij*_ are the fraction of counts of oligotype *j* in that sample, according to:

(3) $$x_{i}=C\left(\left[c_{i1}\;c_{i2}\;\cdots \;c_{\mathrm{iD}}\right]\right)=\frac{\left[c_{i1}\;c_{i2}\;\cdots c_{\mathrm{iD}}\right]}{\sum_{j=1}^{D}c_{\mathrm{ij}}},$$

where C is the closure operation for any vector **c** of *D* positive real components (*D* = 6 in our analysis). If **c**_*i*_ is the vector of counts in sample *i*, then the *n* × *D* compositional data matrix, **X**, can then be constructed as:

(4) $$X=\left(\begin{array}{c} x_{1}\\ x_{2}\\ \vdots \\ x_{n} \end{array}\right)=\left(\begin{array}{c} C\left[c_{1}\right]\\ C\left[c_{2}\right]\\ \vdots \\ C\left[c_{n}\right] \end{array}\right).$$

#### Operations and metrics

Operations and distances analogous to those in Euclidean space can be defined within the simplex. We present here a brief description of those utilized in this manuscript. Analogous to addition is the perturbation operation, ⊕, defined between two compositions as:

(5) $$x⊕y=C\left(\left[x_{1}\cdot y_{1},\;x_{2}\cdot y_{2},\;\cdots \;x_{D}\cdot y_{D}\right]\right).$$

Similarly, perturbation difference is defined as:

(6) $$x⊖y=x⊕y^{-1}=C\left(\left[x_{1}\cdot 1/y_{1},\;x_{2}\cdot \;1/y_{2},\;\cdots x_{D}\cdot 1/y_{D}\right]\right),$$

where the inverse of a composition is defined as:

(7) $$x^{-1}=C\left(\left[1/x_{1},1/x_{2},\cdots 1/x_{D}\right]\right).$$

We also utilize the Aitchison inner product, norm and distance for the simplex (Aitchison, 1986; Pawlowsky‐Glahn *et al*., 2015). The Aitchison inner product is defined as:

(8) $${\left\langle x,y\right\rangle }_{a}=\frac{1}{2D}\sum_{k=1}^{D}\sum_{j=1}^{D}\ln\frac{x_{k}}{x_{j}}\ln\frac{y_{k}}{y_{j}}=\sum_{j=1}^{D}\ln\frac{x_{j}}{g\left(x\right)}\ln\frac{y_{j}}{g\left(y\right)},$$

where *g*(**x**) is the geometric mean across components calculated as:

(9) $$g\left(x\right)={\left(\prod_{j=1}^{D}x_{j}\right)}^{1/D}.$$

The Aitchison norm is defined as:

(10) $${\left\|x\right\|}_{a}=\sqrt{\frac{1}{2D}\sum_{k=1}^{D}\sum_{j=1}^{D}{\left(\ln\frac{x_{k}}{x_{j}}\right)}^{2}}.$$

The squared Aitchison norm divided by number of components,

(11) $$A_{I}^{2}=\frac{1}{D}{\left\|x\right\|}_{a}^{2}=\frac{1}{2D^{2}}\sum_{k=1}^{D}\sum_{j=1}^{D}{\left(\ln\frac{x_{k}}{x_{j}}\right)}^{2},$$

can be used as an index of evenness over the composition, and we refer to $A_{I}^{2}$ as the Aitchison index (Egozcue and Pawlowsky‐Glahn, 2019). This quantity can be scaled as $1-\exp\left(-A_{I}^{2}\right)$ to map between 0 and 1 for comparison to other metrics or indices.

Similarly, Aitchison distance provides a measure of dissimilarity between compositions (Chong and Spencer, 2018):

(12) $$d_{a}\left(x,y\right)={\left\|x⊖y\right\|}_{a}=\sqrt{\frac{1}{2D}\sum_{k=1}^{D}\sum_{j=1}^{D}{\left(\ln\frac{x_{k}}{x_{j}}-\ln\frac{y_{k}}{y_{j}}\right)}^{2}}=\sqrt{\sum_{j=1}^{D}{\left(\ln\frac{x_{j}}{g\left(x\right)}-\ln\frac{y_{j}}{g\left(y\right)}\right)}^{2}}.$$

We calculate the centre of the dataset as:

(13) $$\mathrm{cen}\left(x\right)=\left[g_{1}g_{2}\cdots g_{D}\right],$$

where *g*_*j*_ is the geometric mean of each component, calculated across all samples (as in **X**):

(14) $$g_{j}={\left(\prod_{i=1}^{n}x_{\mathrm{ij}}\right)}^{1/n}.$$

How components covary with each other can be examined with the Aitchison variation matrix, **T** (Pawlowsky‐Glahn *et al*., 2015). Each element of **T** is defined as:

(15) $$\begin{array}{l} t_{\mathrm{kj}}=\mathrm{var}\left(\ln\frac{x_{k}}{x_{j}}\right)\\ t_{\mathrm{kj}}=\frac{1}{n-1}\sum_{i=1}^{n}{\left(\ln\frac{x_{\mathrm{ik}}}{x_{\mathrm{ij}}}-\ln\frac{g_{k}}{g_{j}}\right)}^{2}\;\text{for}\;k,j=1,2,\cdots D, \end{array}$$

where *g*_*j*_ is as Eq. 14. Elements of **T** range from 0 to ∞; low values indicate stronger proportionality between *x*_*k*_ and *x*_*j*_ (a value of 0 indicates the ratio $\frac{x_{k}}{x_{j}}$ is always constant), whereas larger values reflect little proportionality.

#### Visualization

To visualize relationships between components and samples in two dimensions, we construct a compositional biplot (Aitchison and Greenacre, 2002). We perform a singular value decomposition (SVD) on a centred log‐ratio transformed centred data matrix. The centred log‐ratio transformation is defined as:

(16) $$\mathrm{clr}\left(x\right)=\left[\ln\frac{x_{1}}{g\left(x\right)},\ln\frac{x_{2}}{g\left(x\right)},\cdots \ln\frac{x_{D}}{g\left(x\right)}\right],$$

where *g*(**x**) is the geometric mean per sample (Eq. 9). The clr is an isometry between $S^{D}$ and a subspace of ${\mathbb{R}}^{D}$, and has the added benefit of having the same number of components as the original, but dependency within columns (row vectors sum to zero) results in singular covariance matrices (Pawlowsky‐Glahn *et al*., 2015). The inverse clr operation is:

(17) $${\mathrm{clr}}^{-1}\left(x\right)=C\left[\exp\left(x\right)\right].$$

To visualize this high dimensional matrix in two dimensions, we perform an SVD on the matrix **Z**, where:

(18) $$Z=\mathrm{clr}\left(X⊖\mathrm{cen}\left(X\right)\right)=\mathrm{clr}\left(X⊕{\left(\mathrm{cen}\left(X\right)\right)}^{-1}\right),$$

and utilize the first two singular values and corresponding vectors.

#### Zero imputation

Zeros pose a problem for many of the techniques and calculations in compositional data analysis. If a zero in a dataset results from undersampling or detection limits, then it makes sense to replace it with a small value (Pawlowsky‐Glahn *et al*., 2015). We replace zeros in our subcompositions using a Bayesian‐multiplicative treatment described by Martín‐Fernández *et al*. (2015). This method preserves ratios among non‐zero components and zeros are replaced with a posterior Bayesian estimate. Priors are calculated and applied within the following seasons: winter–spring (January 1–June 15), summer (June 16–September 15) and fall (September 16–December 31). These season divisions match those of Hunter‐Cevera *et al*. (2020a), and delineate *Synechococcus* population dynamics, with the exception that winter and spring are combined here due to low number of winter and early spring samples.

#### Isometric log‐ratio transformation

To understand how environmental variables affect the *Synechococcus* composition, we need to be able to examine relationships between environmental variables and relative abundances. As mentioned above, standard statistical analysis is not appropriate for relative data as it does not account for the interdependency among proportions. We utilize the isometric log‐ratio transformation (ilr), and the ‘principle of working in coordinates’ (Pawlowsky‐Glahn *et al*., 2015) to be able to utilize standard multivariate regression.

The ilr is an isometry from $S^{D}$ to ${\mathbb{R}}^{D-1}$ (Pawlowsky‐Glahn *et al*., 2015). Isometric operations preserve distances in the simplex with respect to their counterparts in real space. The transformation produces the coordinates of a composition, $x\in S^{D}$, with respect to an orthonormal basis of $S^{D}$. The ilr transformation is:

(19) $$\mathrm{ilr}\left(x\right)=\left({\left\langle x,e_{1}\right\rangle }_{a},\langle x,e_{2}\rangle_{a},\cdots \langle x,e_{D-1}\rangle_{a}\right),$$

where ⟨**x**, **y**⟩_*a*_ is the Aitchison inner product (Eq. 8). The set of vectors **e**_**i**_, for *i* = 1, 2⋯*D* − 1, forms an orthonormal basis in $S^{D}$ where each **e**_**i**_ is a composition of *D* parts. Vectors are orthonormal in the simplex if

(20) $${\left\langle e_{i},e_{j}\right\rangle }_{a}=0,\text{for}\;i\neq j$$

(21) $${\left\langle e_{i},e_{j}\right\rangle }_{a}=1,\text{for}\;i=j.$$

The ilr transformation is the projection of a composition onto a set of compositional vectors (i.e. they are the coordinates of **x** with respect to a basis in $S^{D}$). This transformation is isometric, and is subcompositionally coherent (analysis of only a portion of the composition is not affected by excluding other components). The projections are real, unbounded values, and can be treated and analysed as real, random variables.

The principle of working in coordinates, developed by Egozcue *et al*. (2003) and Egozcue and Pawlowsky‐Glahn (2005), involves the following set of steps: construct any orthonormal basis, transform the data with this basis, conduct standard multivariate analysis, and then back transform the results to the simplex. In general, the choice of basis should not necessarily matter, but a well‐chosen basis enables interpretation of individual coefficients and parameters on the level of coordinates. A basis based on sequential binary partitions (SBP) within the composition offers an easier and more insightful interpretation than an arbitrary one.

A basis formed from partitions can be developed from expert knowledge or exploratory analysis. We constructed an SBP (Table S4) by analysing the Aitchison variation matrix, **T**. Variation between components can be represented in a dendrogram (Van den Boogaart and Tolosana‐Delgado, 2013), and we use two different clustering algorithms (Fig. S7). Both suggest a close association between O1–O3 and O4–O5 but differ in branches for O2 and O6‐I*. We construct our SBP (Table S4) from these two figures. The first partition separates O2 from the rest of the group (reflecting Fig. S7a). The second partition separates O4, O5 from O1, O3, O6, reflecting the difference in spring and fall relative abundances. Subsequent partitions further divide these two groupings (as in Fig. S7b).

From this SBP, we build an orthonormal basis in $S^{6}$ by use of *balancing* elements. Each balancing element is a vector associated with the k‐th order binary partition, defined as:

(22) $$b_{j}^{k}=\left\{\begin{array}{l} \sqrt{\frac{S}{R\left(R+S\right)}}\;\text{if}\;x_{j}\in r\;\text{group},\\ -\sqrt{\frac{R}{S\left(R+S\right)}}\;\text{if}\;x_{j}\in s\;\text{group},\\ 0,\text{if}\;x_{j}\;\text{is not part of}\;a\;\text{group} \end{array}\right.$$

where *R* is the total number of elements in the *r*‐group and *S* is the total number of elements in the *s*‐group for the *k*th partition. The corresponding balancing elements of the SBP defined in Table S4 are:

(23) $$B=\left(\begin{array}{c} b_{1}\\ b_{2}\\ b_{3}\\ b_{4}\\ b_{5} \end{array}\right)=\left(\begin{array}{cccccc} \frac{1}{\sqrt{30}} & -\frac{\sqrt{5}}{\sqrt{6}} & \frac{1}{\sqrt{30}} & \frac{1}{\sqrt{30}} & \frac{1}{\sqrt{30}} & \frac{1}{\sqrt{30}}\\ \frac{\sqrt{2}}{\sqrt{15}} & 0 & \frac{\sqrt{2}}{\sqrt{15}} & -\frac{\sqrt{3}}{\sqrt{10}} & -\frac{\sqrt{3}}{\sqrt{10}} & \frac{\sqrt{2}}{\sqrt{15}}\\ 0 & 0 & 0 & \frac{1}{\sqrt{2}} & -\frac{1}{\sqrt{2}} & 0\\ \frac{1}{\sqrt{6}} & 0 & \frac{1}{\sqrt{6}} & 0 & 0 & -\frac{\sqrt{2}}{\sqrt{3}}\\ \frac{1}{\sqrt{2}} & 0 & -\frac{1}{\sqrt{2}} & 0 & 0 & 0 \end{array}\right)$$

An orthonormal basis is then constructed with the following operation to **B**:

(24) $$e_{k}=C\left[\exp\left(b_{k}\right)\right]\;\text{for}\;k=1,2,\ldots D-1.$$

The ilr transform is obtained by taking the Aitchison inner product between each observed composition and each vector in the basis (i.e. projecting onto the basis). This calculation reduces to the following direct expression from an SBP to the ilr transform without having to explicitly construct the basis (Pawlowsky‐Glahn *et al*., 2015). For the *k*th SBP:

(25) $${\mathrm{ilr}}_{k}\left(x_{i}\right)=\sqrt{\frac{\mathrm{RS}}{R+S}}\ln\left[\frac{{\left(\prod_{w=1}^{R}r_{w}\right)}^{1/R}}{{\left(\prod_{q=1}^{S}s_{q}\right)}^{1/S}}\right]\;\text{for}\;k=1\ldots D-1$$

where **r** and **s** denote the compositions composed of elements only belonging to either the *r* or *s* group respectively with counters *w* and *q*, for each *k* partition. These equations illustrate the fact that this transformation is a log ratio of groups of components. The term balancing element also becomes clear; it provides a measure of the relative importance of one group against the other through means of the exponent. For the SBP in Table S4, we obtain the following formulas for the ilr transformation:

(26) $${\mathrm{ilr}}_{1}\left(x_{i}\right)=\ln\left[\frac{{\left(x_{i1}\cdot x_{i3}\cdot x_{i4}\cdot x_{i5}\cdot x_{i6}\right)}^{\sqrt{1/30}}}{{\left(x_{i2}\right)}^{\sqrt{5/6}}}\right]$$

(27) $${\mathrm{ilr}}_{2}\left(x_{i}\right)=\ln\left[\frac{{\left(x_{i1}\cdot x_{i3}\cdot x_{i6}\right)}^{\sqrt{2/15}}}{{\left(x_{i4}\cdot x_{i5}\right)}^{\sqrt{3/10}}}\right]$$

(28) $${\mathrm{ilr}}_{3}\left(x_{i}\right)=\ln\left[\frac{{\left(x_{i4}\right)}^{\sqrt{1/2}}}{{\left(x_{i5}\right)}^{\sqrt{1/2}}}\right]$$

(29) $${\mathrm{ilr}}_{4}\left(x_{i}\right)=\ln\left[\frac{{\left(x_{i1}\cdot x_{i3}\right)}^{\sqrt{1/6}}}{{\left(x_{i6}\right)}^{\sqrt{2/3}}}\right]$$

(30) $${\mathrm{ilr}}_{5}\left(x_{i}\right)=\ln\left[\frac{{\left(x_{i1}\right)}^{\sqrt{1/2}}}{{\left(x_{i3}\right)}^{\sqrt{1/2}}}\right],$$

where *x*_*ij*_ is the proportion of each *j* oligotype for sample *i* (relative to the subcomposition of O1–O6). Transformation from ilr coordinates back to compositions is achieved with the inverse ilr operation:

(31) $$y=\mathrm{ilr}\left(x\right)$$

(32) $${\mathrm{ilr}}^{-1}\left(y\right)=C\left[\exp\left(y\right)\cdot B\right],$$

where **B** is the contrast matrix (Eq. 23).

### Multivariate regression

Transformed compositions (i.e. coordinates or log contrasts) served as response variables in multivariate regression, with predictor variables as temperature, weekly averaged light, nitrate+nitrite, phosphate, ammonium and silicate. Because compositions may reflect integrated light over some time, we used the average light level of the week prior to sampling as the variable (rather than light on day of sampling). We note though that we do not have detailed information on the light levels experienced at depth; significant attenuation of light can occur with an increase in eukaryotic phytoplankton [Sosik unpublished data, Hunter‐Cevera *et al*. (2020a)]. Relationships between coordinates and some environmental parameters did not appear linear (Fig. 3A,B,F,G), and we chose to fit and evaluate coordinates within seasons, separately. Seasons were delineated as winter–spring (January 1–June 15), summer (June 16–September 15) and fall (September 16–December 31), the same as that for zero imputation. These seasons match delineations for different *Synechococcus* growth dynamics (Hunter‐Cevera *et al*., 2020a).

We fit a standard multivariate linear model following that of Rencher (2002) for data belonging to each season (winter/spring, summer and fall). We used a forward step selection method to determine which variables should be included in the model. At each round, we tested the significance of one candidate variable by constructing Wilk's lambda, Λ, from the ratio of Λ for the full and reduced models. We calculate *p*‐values using the F‐distribution approximation. Please see chapters 6 and 10 of Rencher (2002) for more details.

Fitted parameters values provide information on how each of the ilr coordinates varies within season. In addition to examining these parameters, we also find insight from the transformation of parameters back to the simplex (Table 2). Regression parameters are transformed to compositions with the ilr^−1^ calculation (Eq. 32) and the original balance (Eq. 23). Interpretations of parameter compositions are slightly different and we refer to Van den Boogaart and Tolosana‐Delgado (2013). These authors describe the intercept as the expected composition if variable values were zero [which is not a realistic environmental situation in our case (i.e. temperature = 0°C and radiation = 0 MJ m^−2^)]. The transformed slope parameters are interpreted as the perturbation applied to a composition if variables increase by one unit.

All compositional data analysis and multivariate regression were performed in Julia (v 1.2.0), with the exception of Fig. S8, which was produced with the ‘compositions’ package in R (Van den Boogaart and Tolosana‐Delgado, 2013).

## Data Availability

Unmerged and unfiltered sequence reads are available at NCBI under BioProject ID PRJNA725036. Merged, filtered and taxonomically identified sequences are available on the MBL VAMPS website at vamps2.mbl.edu, under project MVCO_2010_2018_timeseries. Details of sequencing analysis and processing pipeline, including scripts, bash commands and full primer sequences are available at github.com/hsosik/NES-LTER/tree/master/amplicon_sequencing/V6V8. Compositional data analysis code is available at github.com/khuntercevera/coda_utilities/.

## Supporting information

**FigureS1:** Comparison between observed and expected proportions of Synechococcus oligotypes for two mock communities. Color indicates strain as labeled in expected column. Replicate D in community 2 was processed from a disk lter; all others utilized Sterivex cartridges.
**Figure S2:** Relationship between proportion of Synechococcus reads (of total reads) and Synechococcus concentration per sample at MVCO displayed in A) linear and B) log scale.
**Figure S3**: Heat map illustrating base pair mistmatches among the V6‐V8 region of dierent unique clade representative sequences and MVCO oligotype sequences. Sequence labels match those in Table S3 and Table S6.
**Figure S4:** Proportions of oligotypes and other Synechococcus sequences (aggregate of oligotypes 7‐14 and unclassied sequences) for environmental samples for which amplication and sequencing replicates exist. Color indicates oligotype as indicated in color bar. Number of Synechococcus sequences per sample is denoted to the right of each bar. For sample 2018‐09‐05, note that this sample was processed both with a Sterivex lter cartridge and PES disk lter and indicated on the axis label, and is therefore not a true duplicate, but rather a comparison of lters.
**Figure S5:** Relative abundance of less abundant oligotypes (O7 ‐ O14) at MVCO.
**Figure S6:** Heat map illustrating dissimilarity between dierent seasonal samples. Color represents Aitchison distance calculated between each sample (Eqn. 12). Samples are grouped by season and appear in order of year day to highlight similarities within and dierences among seasons.
**Figure S7:** Dendrograms formed with Aitchison variation as distance with two dierent clustering methods.
**Figure S8:** Coda‐dendrograms as according to Van den Boogart and Tolosana‐Delgado (2013) and Pawlowsky‐Glahn et al. (2015) for samples belonging to each season. Figures all have same partitioning (as in Fig. S7), but dier in segment join location and segment lengths. Coordinate mean is the center bar on the segments joining two partitions. Boxes on segments indicate quantiles of coordinate values. Line lengths indicate coordinate variance.
**Figure S9:** Relationship between ilr coordinates and nutrients at MVCO: phosphate (top panels), silicate (second panels), ammonium (third panels) and nitrate+nitrite (bottom panels). Color indicates season and year day. The zero line is indicated in each plot for reference.
**Figure S10:** A1‐A5) Time series of ilr coordinates and corresponding mulitvariate regression model ts for winter/spring (blue dots), summer (orange dots), and fall (green dots). B1‐B5) Same as in A1‐A5, except data is plotted by year day. Relationships between ilr coordinates and temperature (C1‐C5) and weekly‐averaged radiation (D1‐D5), with model ts indicated by colors as in A panels.

**Table S1:** Synechococcus strains (and corresponding clade) used to construct mock communities.
**Table S2:** Total merged reads and reads identied as Synechococcus for each replicate of the mock communities.
**Table S3:** Read count and clade/subclade matches (or closest match) for each oligotype at MVCO.
**Table S4:** Sequential binary partition for the composition consisting of the six most abundant Syne‐chococcus oligotypes (O1‐O6). Each row indicates a partition (denoted by k). Partition groups, either r or s, are denoted by square brackets in the second panel, and how each oligotype is assigned to a group is denoted in the third panel (note that not all partitions contain all oligotypes). The number of elements belonging to each group for each partition are listed in last panel.
**Table S5:** Wilk's lambda and p‐values for additional environmental variables tested in multivariate linear regression. Lambda values are constructed from a full model compared to a reduced model. Full model includes the variables listed in the reduced column plus one additional variable (listed in full model column)
**TableS6:** Separate le: Database of Synechococcus strains used to infer clade or subclade identity of oligotypes. Columns include clade, strain name, Genbank accession number, source reference, length of V6‐V8 region, and corresponding within‐clade, unique V6‐V8 sequence designation as in Fig. S3.
