# Supplementary material for: Seasonal environmental variability drives microdiversity within a coastal Synechococcus population
Source: Environ Microbiol. 2021 Jul 26;23(8):4689–705. doi: 10.1111/1462-2920.15666 (PMC8456951; doi:10.1111/1462-2920.15666)
Supplement: Supplementary file 1 — FigureS1: Comparison between observed and expected proportions of Synechococcus oligotypes for two mock communities. Color indicates strain as labeled in expected column. Replicate D in community 2 was processed from a disk lter; all others utilized Sterivex cartridges. Figure S2: Relationship between proportion of Synechococcus reads (of total reads) and Synechococcus concentration per sample at MVCO displayed in A) linear and B) log scale. Figure S3: Heat map illustrating base pair mistmatches among the V6‐V8 region of dierent unique clade representative sequences and MVCO oligotype sequences. Sequence labels match those in Table S3 and Table S6. Figure S4: Proportions of oligotypes and other Synechococcus sequences (aggregate of oligotypes 7‐14 and unclassied sequences) for environmental samples for which amplication and sequencing replicates exist. Color indicates oligotype as indicated in color bar. Number of Synechococcus sequences per sample is denoted to the right of each bar. For sample 2018‐09‐05, note that this sample was processed both with a Sterivex lter cartridge and PES disk lter and indicated on the axis label, and is therefore not a true duplicate, but rather a comparison of lters. Figure S5: Relative abundance of less abundant oligotypes (O7 ‐ O14) at MVCO. Figure S6: Heat map illustrating dissimilarity between dierent seasonal samples. Color represents Aitchison distance calculated between each sample (Eqn. 12). Samples are grouped by season and appear in order of year day to highlight similarities within and dierences among seasons. Figure S7: Dendrograms formed with Aitchison variation as distance with two dierent clustering methods. Figure S8: Coda‐dendrograms as according to Van den Boogart and Tolosana‐Delgado (2013) and Pawlowsky‐Glahn et al. (2015) for samples belonging to each season. Figures all have same partitioning (as in Fig. S7), but dier in segment join location and segment lengths. Coordinate mean is the center bar on the segments [file EMI-23-4689-s002.pdf]

Supplementary Information

Full length 16S sequencing of MVCO strains

Approximately full length 16S sequences were previously obtained for some of the strains isolated from MVCO (accession numbers KU867918.1-KU867949.1, (Hunter-Cevera 2014)). Unfortunately, these 32 sequences do not span the entirety of the V6-V8 region due to the primer set used (107F and 1313R cyanobacteria-specific 16S rRNA primers, (Fuller et al. 2003)). We re-sequenced the 16S rRNA gene for some of these strains in addition to 4 new strains (see below isolation procedure). Approximately 2 mL of dense culture isolate was centrifuged at 10,000 rpm for 6 minutes to pellet cells. DNA was extracted with a DNeasy UltraClean Microbial Kit (Qiagen), following manufacturer's instructions with the exception of final elution volume (75 μL). Approximately 30 ng of DNA was added to PCR reactions with general primer set 27F: 5'-AGA-GTT-TGA-TCM-TGG-CTC-AG-3' and 1492R: 5'-TAC-GGY-TAC-CTT-GTT-ACG-ACT-T-3' or cyanobacteria forward primer 107F: 5-GGA-CGG-GTG-AGT-AAC-GCG-TG-3' and the modified general bacterial reverse primer 1518R-mod: 5'-AGG-AGG-TGA-TCC-ANC-CRC-A -3'. Reagent concentrations and cycling conditions were the same for each primer set. Final primer concentration was 0.2 μM in a total reaction volume of 50 μL with 1.5 mM MgCl2, 0.2 mM dNTPs in 1x AmpliTaq Gold 360 Buffer with 1 unit of Amplitaq Gold polymerase. Reactions were performed with an initial denaturation period of 10 min at 95 °C; followed by 33 cycles of 30 seconds at 95 °C, 30 seconds at 55 °C, 90 seconds at 72 °C; and then a final extension step at 72 °C for 5 min. Product was cleaned by standard ethanol precipitation. Cleaned products were cloned into TOPO vectors for sequencing (TOPO TA Kit, Invitrogen) and transformed into chemically competent *E. coli* TOP10 cells (Invitrogen) following manufacturer's instructions. At least 10 positive colonies (determined by selection on kanamycin LB plates) were picked. Plasmids were obtained via PureLink Quick Plasmid Miniprep Kit (Invitrogen). Plasmids were sequenced at the University of Chicago Comprehensive Cancer Center DNA Sequencing Facility. Two sequencing reactions per plasmid were performed using M13 forward and reverse primers. Primer and vector sequences were removed and chromatogram visually checked for quality and base calls, and merged in BioEdit (Hall 1999). For each *Synechococcus* strain, 5-10 sequences were aligned to check for sequencing error and form a consensus sequence if needed. Sequences were identified by BLAST search against 16S rRNA accessions in NCBI Genbank. Resulting sequences have been uploaded to Genbank and can be accessed with the numbers:

MT994346-MT994359, and MW988106-MW988107 (see Table S6 for more details).

Isolation of additional MVCO strains

Enrichments for *Synechococcus* were started from MVCO surface seawater samples in summer and fall 2017 as described in Hunter-Cevera et al. (2016). Dilution to extinction enrichments were also prepared; surface seawater was diluted with filtered seawater (0.22 μm) to 10^3, 10^4, and 10^5-fold dilution. All tubes were allowed to incubate in low-medium light levels ($\sim$ 75 μmol m^{-2} s^{-1}). We isolated single colonies from enrichments as described in Hunter-Cevera et al. (2016) with agar plates. Once an isolate was stable and could be grown to appreciable mass, clade identity was found by sequencing the *petB* phylogenetic marker (Mazard et al. 2012). DNA was extracted with a DNeasy microbial kit following manufacturer's instructions (Qiagen). DNA purity and concentration were checked on a nanodrop spectrophotometer (ThermoScientific). The *petB* gene was PCR amplified with primers PetB2-F (5'-ACT-GGT-TCV-ASG-ARC-GTC-T-3') and PetB2-R (5'-CCY-TGC-TTV-CGR-ATC-ATC-AGG-3') (Mazard et al. 2012). Reaction mixtures of 50 μL consisted of 0.4 μM of each primer, 1.5 mM MgCl2, 0.2 mM dNTPs in 1x AmpliTaq Gold 360 Buffer with 1 unit of Amplitaq Gold polymerase, and 30 ng of isolate DNA. Cycle conditions consisted of a denaturation step of 10 min at 95 °C, followed by 35 cycles of 30 seconds at 94 °C, 30 seconds at 55 °C, and 60 seconds at 72 °C, and a final extension step of 6 minutes at 72 °C. Reactions were performed on a Veriti Thermal Cycler (Applied Biosystems). PCR product was cleaned following standard ethanol precipitation and final concentrations were checked on a NanoDrop spectrophtometer (ThermoFisher). Products were sequenced at the University of Chicago Comprehensive Cancer Center DNA Sequencing Facility with PetB2-F primer. Primer and vector sequences were removed and chromatogram visually checked for quality and base calls in BioEdit (Hall 1999). Phylogenetic reconstructions were performed in ARB (Ludwig et al. 2004), and clade identity was assigned based on closest phylogenetic grouping.

References

Fuller, N. J., D. Marie, F. Partensky, D. Vaulot, A. F. Post, and D. J. Scanlan. 2003. Clade-specific 16S ribosomal DNA oligonucleotides reveal the predominance of a single marine *Synechococcus* clade throughout a stratified water column in the Red Sea. Applied and Environmental Microbiology, 69(5):2430–2443.

Hall, T. A. 1999. BioEdit: a user-friendly biological sequence alignment editor and analysis program for Windows 95/98/NT. Nucleic Acids Symposium Series, 41:95–98.

Hunter-Cevera, K. R. 2014. Population dynamics and diversity of *Synechococcus* on the New England Shelf. Ph.D. thesis, Massachusetts Institute of Technology and Woods Hole Oceanographic Institution.

Hunter-Cevera, K. R., A. F. Post, E. E. Peacock, and H. M. Sosik. 2016. Diversity of *Synechococcus* at the Martha's Vineyard Coastal Observatory: Insights from culture isolations, clone libraries, and flow cytometry. Microb. Ecol., 71(2):276–289.

Ludwig, W., O. Strunk, R. Westram, L. Richter, H. Meier, A. Buchner, T. Lai, S. Steppi, G. Jobb, W. Förster, et al. 2004. ARB: a software environment for sequence data. Nucleic Acids Research, 32(4):1363–1371.

Mazard, S., M. Ostrowski, F. Partensky, and D. J. Scanlan. 2012. Multi-locus sequence analysis, taxonomic resolution and biogeography of marine *Synechococcus*. Environmental Microbiology, 14(2):372–386.

Pawlowsky-Glahn, V., J. J. Egozcue, and R. Tolosana-Delgado. 2015. Modeling and Analysis of Compositional Data. Wiley.

Van den Boogaart, K. G. and R. Tolosana-Delgado. 2013. Analyzing compositional data with R, volume 122. Springer.

Supplementary Figures

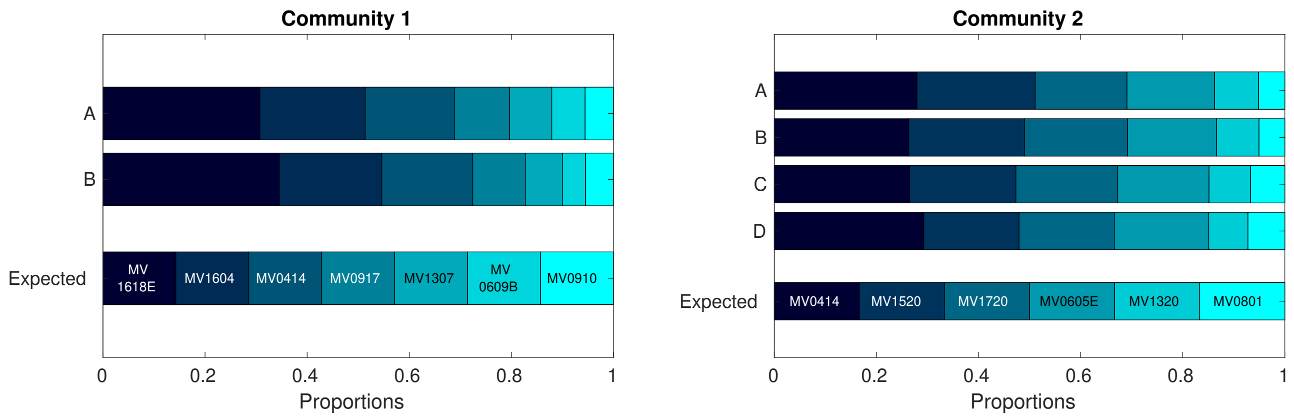

Figure S1: Comparison between observed and expected proportions of *Synechococcus* oligotypes for two mock communities. Color indicates strain as labeled in expected column. Replicate D in community 2 was processed from a disk filter; all others utilized Sterivex cartridges.

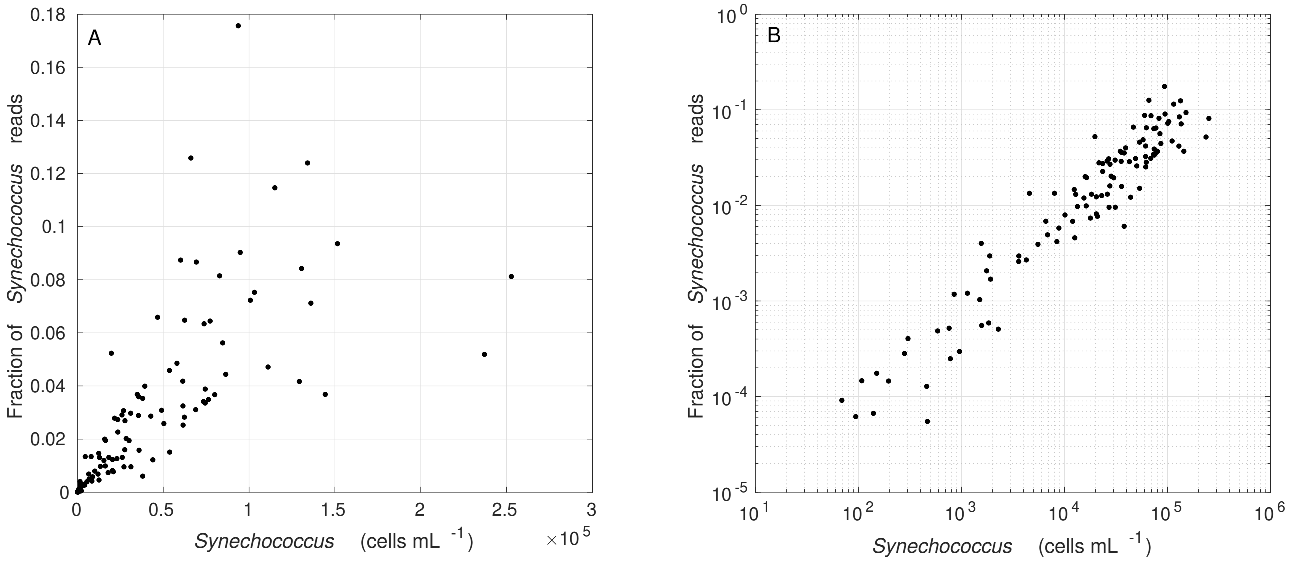

Figure S2: Relationship between proportion of *Synechococcus* reads (of total reads) and *Synechococcus* concentration per sample at MVCO displayed in A) linear and B) log scale.

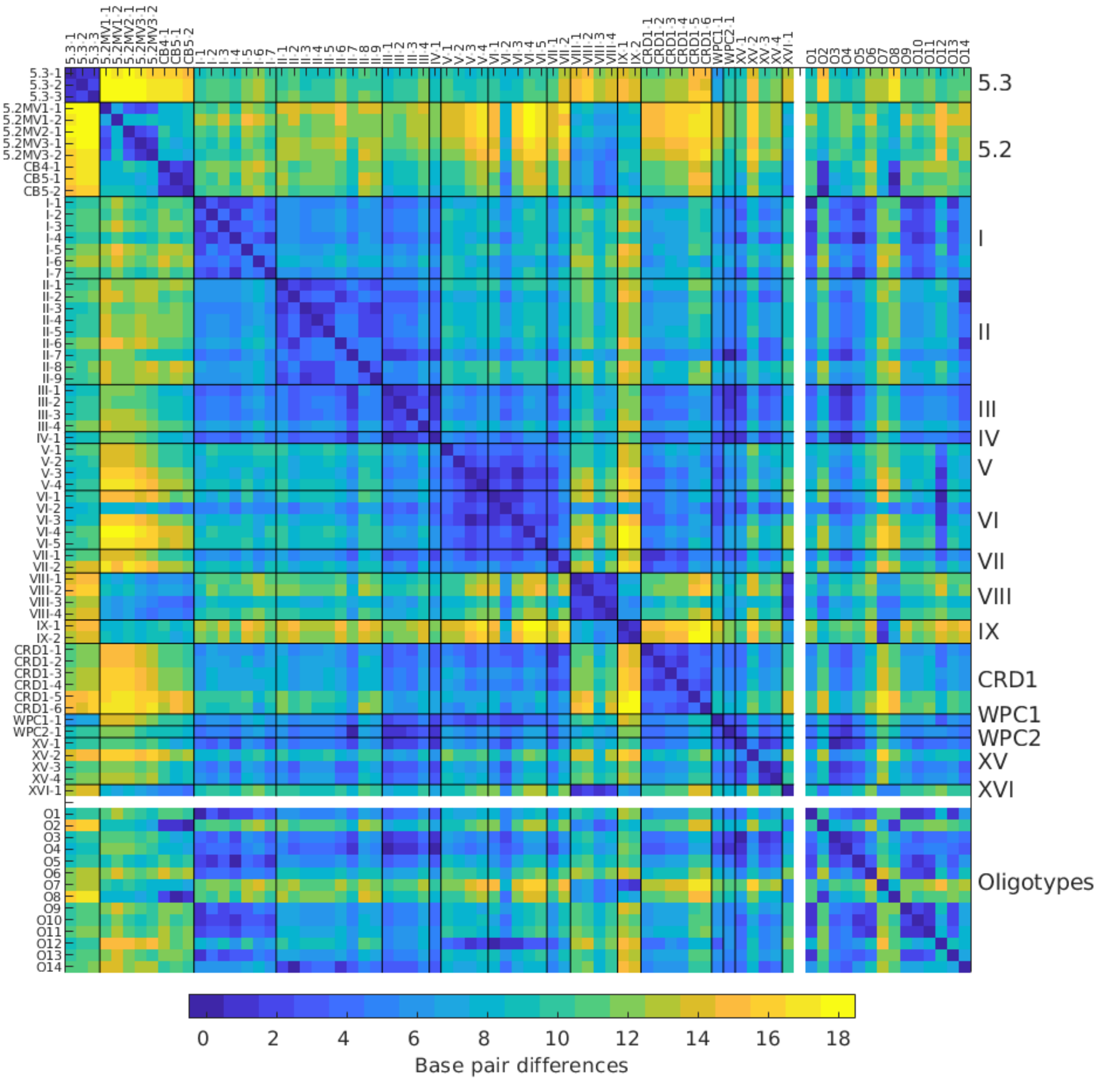

Figure S3: Heat map illustrating base pair mistmatches among the V6-V8 region of different unique clade representative sequences and MVCO oligotype sequences. Sequence labels match those in Table S3 and Table S6.

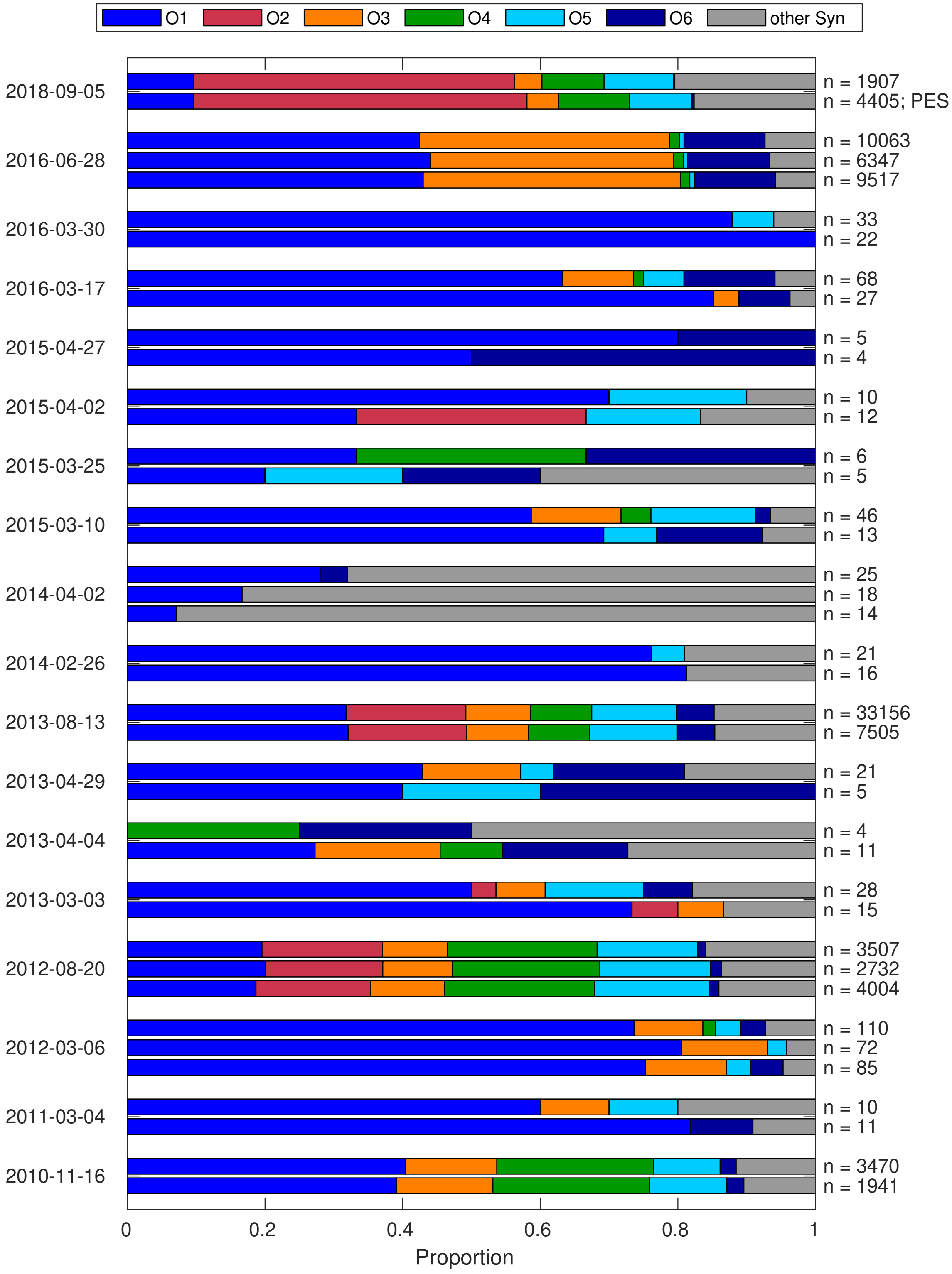

Figure S4: Proportions of oligotypes and other *Synechococcus* sequences (aggregate of oligotypes 7-14 and unclassified sequences) for environmental samples for which amplification and sequencing replicates exist. Color indicates oligotype as indicated in color bar. Number of *Synechococcus* sequences per sample is denoted to the right of each bar. For sample 2018-09-05, note that this sample was processed both with a Sterivex filter cartridge and PES disk filter and indicated on the axis label, and is therefore not a true duplicate, but rather a comparison of filters.

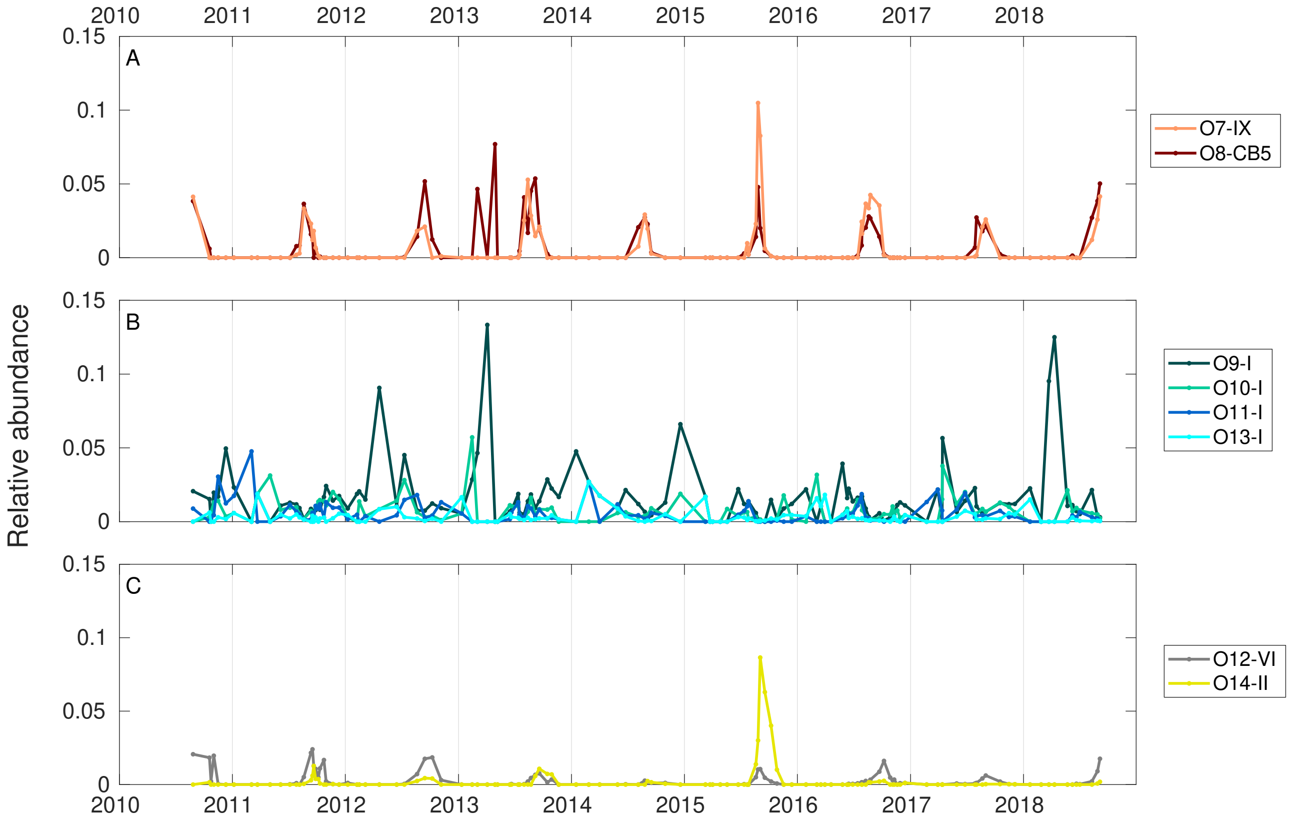

Figure S5: Relative abundance of less abundant oligotypes (O7 - O14) at MVCO.

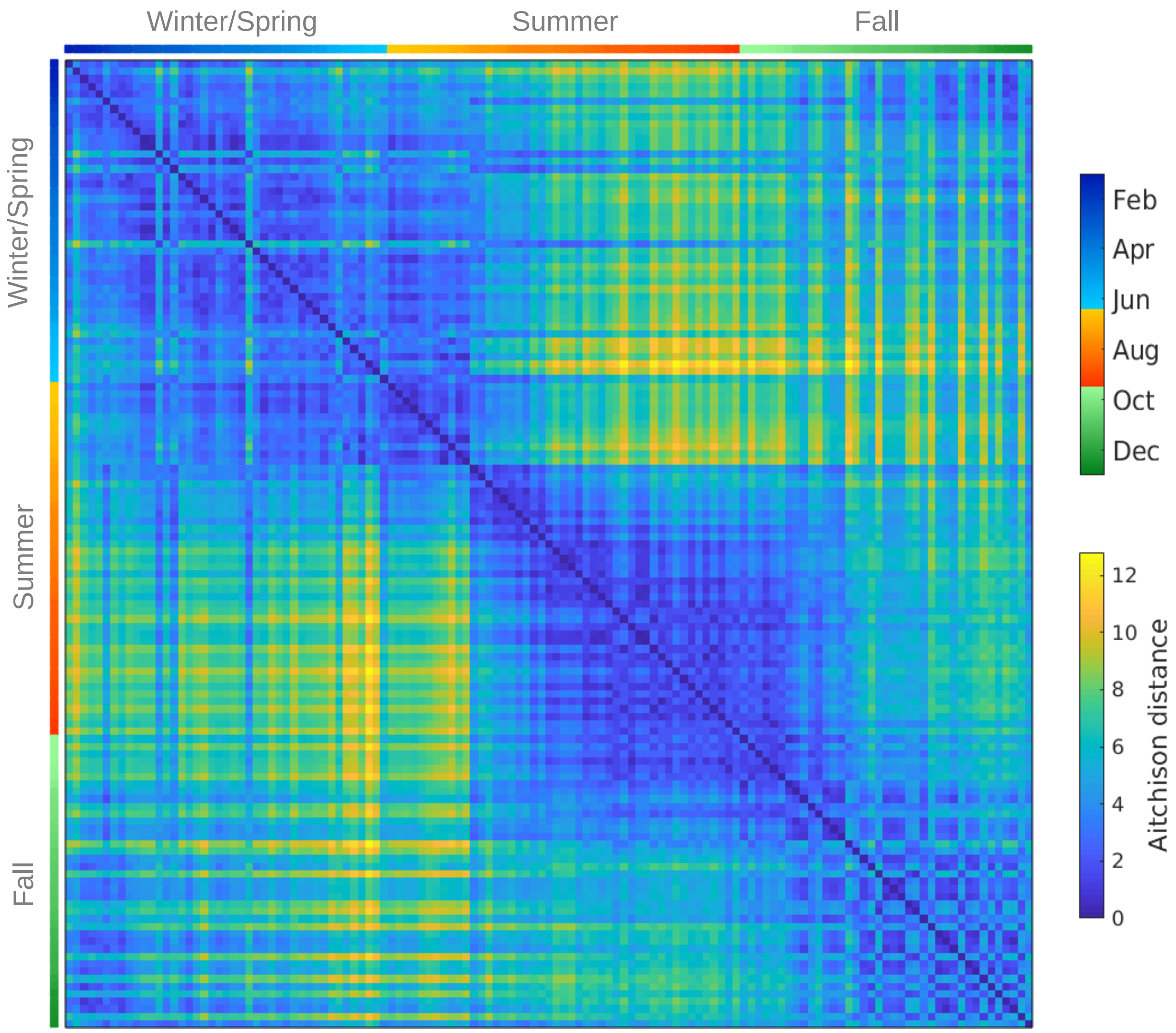

Figure S6: Heat map illustrating dissimilarity between different seasonal samples. Color represents Aitchison distance calculated between each sample (Eqn. 12). Samples are grouped by season and appear in order of year day to highlight similarities within and differences among seasons.

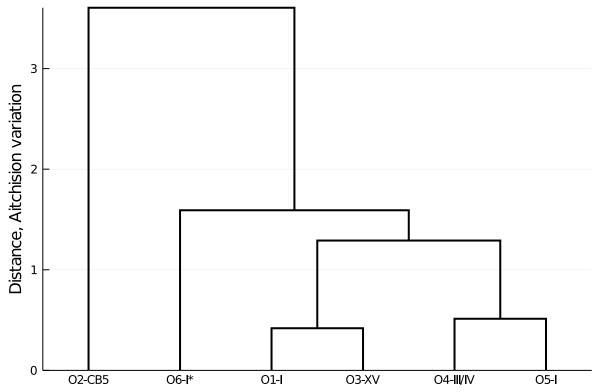

(a) Clustering performed with single linkage (minimum distance between any of the cluster members).

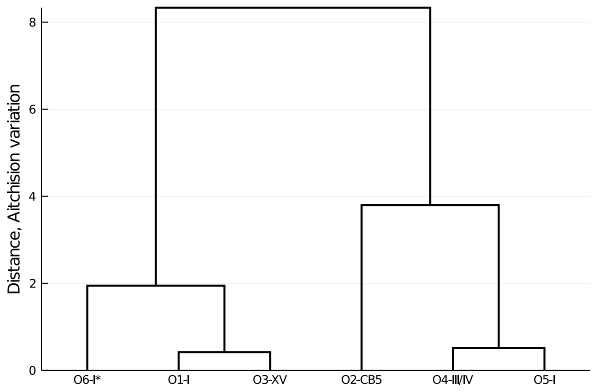

(b) Clustering performed with complete linkage (maximum distance between any of the members)

Figure S7: Dendrograms formed with Aitchison variation as distance with two different clustering methods.

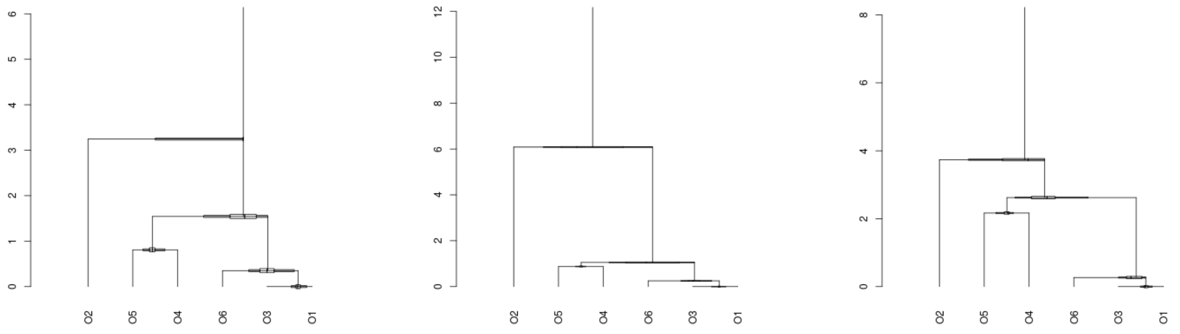

(a) Winter/Spring samples

(b) Summer samples

(c) Fall samples

Figure S8: Coda-dendrograms as according to Van den Boogart and Tolosana-Delgado (2013) and Pawlowsky-Glahn et al. (2015) for samples belonging to each season. Figures all have same partitioning (as in Fig. S7), but differ in segment join location and segment lengths. Coordinate mean is the center bar on the segments joining two partitions. Boxes on segments indicate quantiles of coordinate values. Line lengths indicate coordinate variance.

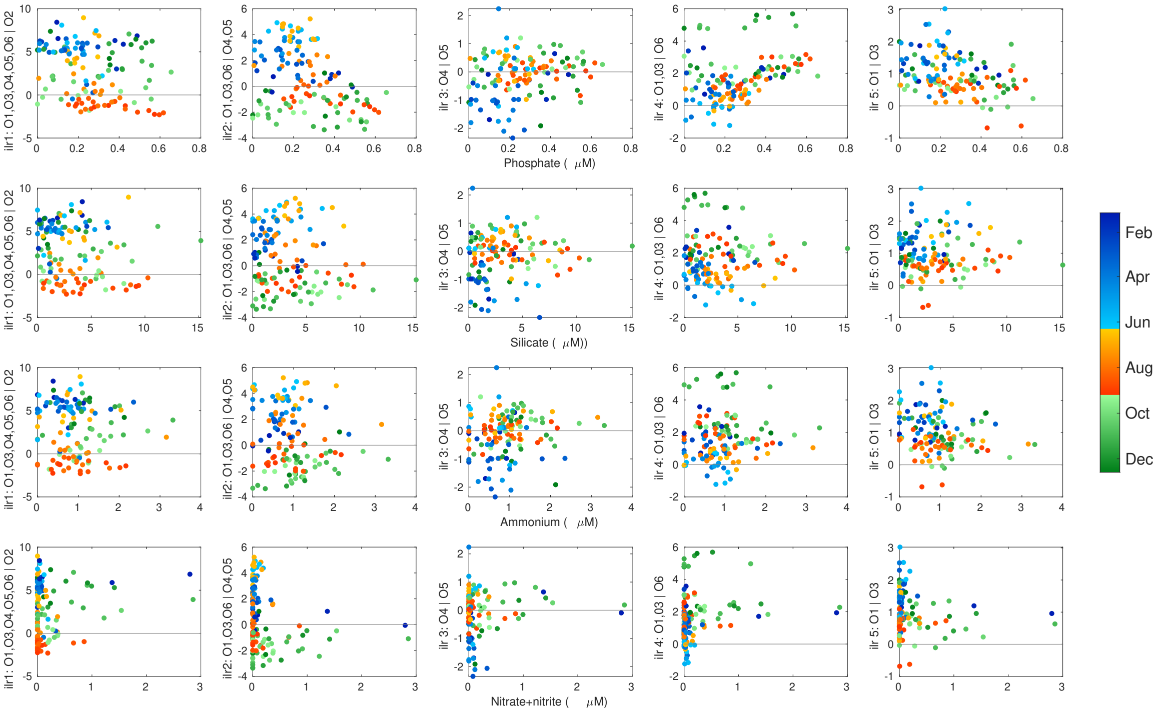

Figure S9: Relationship between ilr coordinates and nutrients at MVCO: phosphate (top panels), silicate (second panels), ammonium (third panels) and nitrate+nitrite (bottom panels). Color indicates season and year day. The zero line is indicated in each plot for reference.

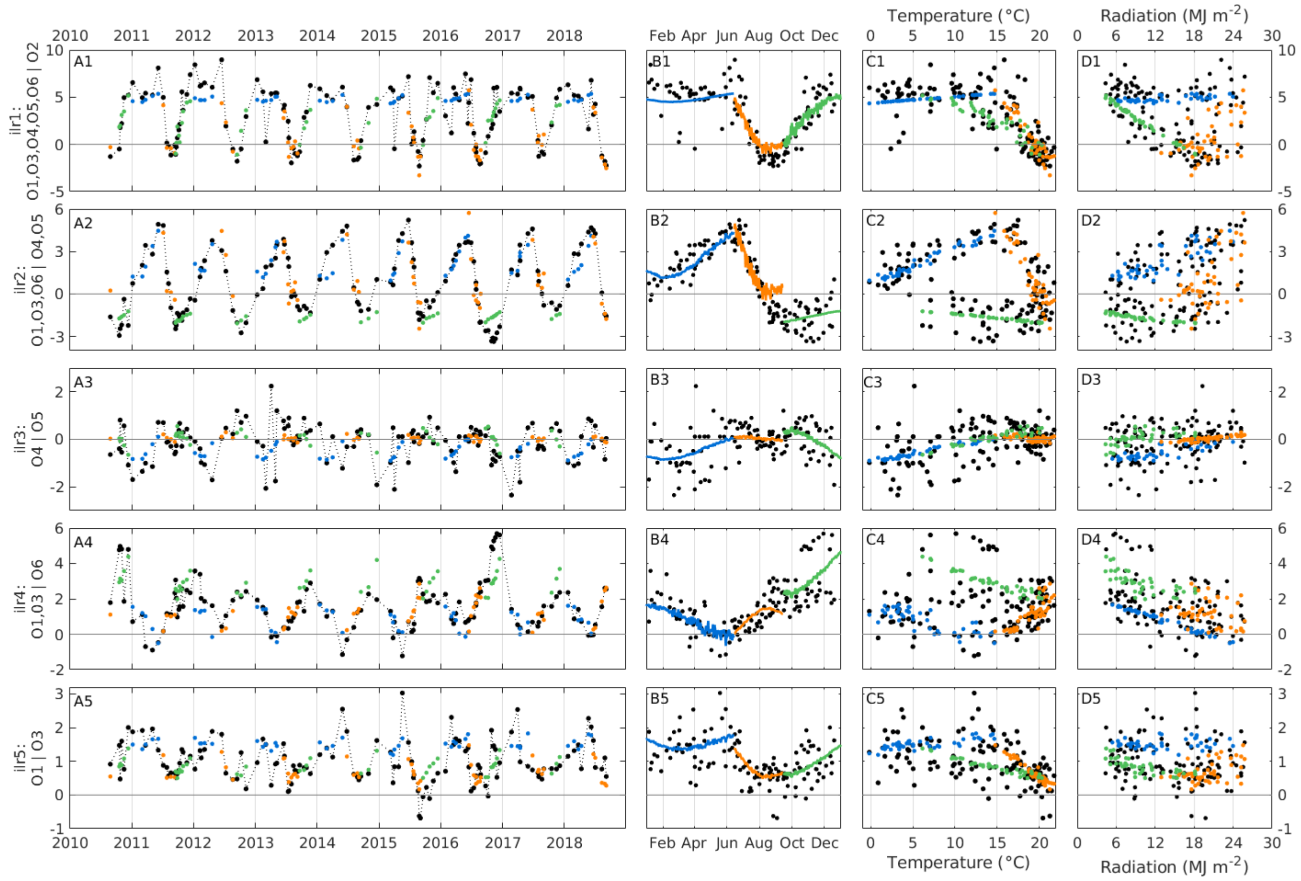

Figure S10: A1-A5) Time series of ilr coordinates and corresponding mulitvariate regression model fits for winter/spring (blue dots), summer (orange dots), and fall (green dots). B1-B5) Same as in A1-A5, except data is plotted by year day. Relationships between ilr coordinates and temperature (C1-C5) and weekly-averaged radiation (D1-D5), with model fits indicated by colors as in A panels.

Supplementary Tables

Table S1: *Synechococcus* strains (and corresponding clade) used to construct mock communities.

Mock Community 1		Mock Community 2	
Clade	Strain	Clade	Strain
I, subclade C	MV1917	I, subclade C	MV0801
I, subclade E	MV1307	I, subclade E	MV1320
CB5	MV0414	CB5	MV0414
VI	MV1618E	CB5	MV060E
II	MV1604B	II	MV1720
VII	MV0609B	III	MV1520
MV5.21	MV0910		

Table S2: Total merged reads and reads identified as *Synechococcus* for each replicate of the mock communities.

Community/Replicate	Total Reads	*Synechococcus* Reads
1: Replicate A	66,702	11,539
1: Replicate B	91,956	6,090
2: Replicate A	34,158	28,010
2: Replicate B	15,316	12,851
2: Replicate C	92,992	7,8571
2: Replicate D	6,823	5,827

Table S3: Read count and clade/subclade matches (or closest match) for each oligotype at MVCO.

Oligotype	Read count	Direct clade match	Closest clade match	Corresponding V6-V8 seq.
O1	108,124	I	—	I-1
O2	44,995	CB5	—	CB5-2
O3	41,226	XV	—	XV-1
O4	35,586	III, IV	—	III-1, IV-1
O5	32,391	I	—	I-4
O6	21,956	—	I	3 bp from I-1, I-4
O7	5583	—	IX	1 bp from IX-2
O8	3898	CB5	—	CB5-I
O9	3631	—	I	1 bp from I-1
O10	2321	—	I	1 bp from I-1, I-4
O11	2099	—	I	2 bp from I-1, I-4
O12	965	VI	—	VI-I
O13	806	—	I	1 bp from I-1
O14	771	II	—	II-2

Table S4: Sequential binary partition for the composition consisting of the six most abundant *Synechococcus* oligotypes (O1-O6). Each row indicates a partition (denoted by k). Partition groups, either **r** or **s**, are denoted by square brackets in the second panel, and how each oligotype is assigned to a group is denoted in the third panel (note that not all partitions contain all oligotypes). The number of elements belonging to each group for each partition are listed in last panel.

k	**r**	**s**	O_1	O_2	O_3	O_4	O_5	O_6	R	S
1	$[O_1, O_3, O_4, O_5, O_6]$	$[O_2]$	r	s	r	r	r	r	5	1
2	$[O_1, O_3, O_6]$	$[O_4, O_5]$	r	0	r	s	s	r	3	2
3	$[O_4]$	$[O_5]$	0	0	0	r	s	0	1	1
4	$[O_1, O_3]$	$[O_6]$	r	0	r	0	0	s	2	1
5	$[O_1]$	$[O_3]$	r	0	s	0	0	0	1	1

Table S5: Wilk's lambda and p-values for additional environmental variables tested in multivariate linear regression. Lambda values are constructed from a full model compared to a reduced model. Full model includes the variables listed in the reduced column plus one additional variable (listed in full model column).

Season	Reduced Model	Full model	Λ	p-value
Winter/Spring	Temperature + weekly-avg. light	Ammonium	0.990	0.996
	Temperature + weekly-avg. light	Nitrate+nitrite	0.863	0.355
	Temperature + weekly-avg. light	Phosphate	0.779	0.096
	Temperature + weekly-avg. light	Silicate	0.767	0.078
Summer	Temperature + weekly-avg. light + phosphate	Ammonium	0.820	0.179
	Temperature + weekly-avg. light + phosphate	Nitrate+nitrite	0.905	0.572
	Temperature + weekly-avg. light + phosphate	Silicate	0.769	0.073
Fall	Temperature + weekly-avg. light	Ammonium	0.738	0.064
	Temperature + weekly-avg. light	Nitrate+nitrite	0.820	0.233
	Temperature + weekly-avg. light	Phosphate	0.851	0.352
	Temperature + weekly-avg. light	Silicate	0.898	0.592

Table S6: Separate file: Database of *Synechococcus* strains used to infer clade or subclade identity of oligotypes. Columns include clade, strain name, Genbank accession number, source reference, length of V6-V8 region, and corresponding within-clade, unique V6-V8 sequence designation as in Fig. S3.
